# Causes of Excess Deaths in the US Compared With Other High-Income Countries

**DOI:** 10.1001/jamanetworkopen.2026.6147

**Published:** 2026-05-08

**Authors:** Jacob Bor, Rafeya V. Raquib, David Himmelstein, Steffie Woolhandler, Andrew C. Stokes

**Affiliations:** 1 Department of Global Health, Boston University School of Public Health, Boston, Massachusetts; 2 Department of Epidemiology, Boston University School of Public Health, Boston, Massachusetts; 3 Department of Medicine, Harvard Medical School, Boston, Massachusetts; 4 Hunter College, City University of New York, New York

## Abstract

**Importance:**

The US has higher mortality rates than other high-income countries (HICs). However, a comprehensive analysis of excess US deaths encompassing all leading causes of death over the past 2 decades is currently lacking.

**Objective:**

To investigate causes of death responsible for excess US mortality compared with other HICs and how the causes of death involved in this US mortality disadvantage have changed over time.

**Design, Setting, and Participants:**

This repeated cross-sectional study included mortality data from the World Health Organization Mortality Database spanning 1999 to 2022 for the US and 17 other HICs. Data were analyzed from September 2023 to December 2025.

**Exposures:**

Residing in the US vs another of the included HICs.

**Main Outcome and Measures:**

The main outcome was excess US mortality in each year due to specific causes of death. Differences between the US and other HICs were quantified for each cause of death as (1) excess US deaths (ie, absolute difference between observed deaths and deaths expected if US death rates equaled the rates of other HICs); (2) years of life lost (YLL) resulting from excess US deaths; and (3) mortality rate ratios (ie, ratios of observed to expected age-standardized death rates).

**Results:**

A total of 63 547 318 deaths occurred in the US from 1999 to 2022 (50.4% among males; mean [SD] age at death, 73.2 [18.5] years). In this period, 12 675 646 excess US deaths occurred, increasing from 346 166 in 1999 to 905 159 in 2022. Circulatory diseases were the leading cause of excess US deaths every year except 2010, increasing after 2001 for ages 45 to 64 years and after 2009 for ages 65 years or older. Together, circulatory and metabolic diseases accounted for 52% of excess US deaths in 2022. Excess US deaths due to drug poisonings, alcohol, and suicide increased from −5762 in 1999 to 131 151 in 2022; together, these 3 causes accounted for 24% of the increase in excess US deaths overall and most of the increase in excess US deaths for individuals aged 0 to 44 years. In 2022, deaths from drug poisonings were 7.48 times higher in the US than in other HICs. In 2020 and 2021, 19% and 23% of excess US deaths, respectively, were attributed to COVID-19, but excess US deaths from other causes also increased.

**Conclusions and Relevance:**

In this repeated cross-sectional study of cross-national mortality, the US had substantially higher death rates than other HICs between 1999 and 2022, despite having similar access to advanced medical technology. Many of these excess US deaths could likely be avoided by adopting health and social policies that have benefited other HICs. These descriptive findings should be interpreted in light of uncertainty arising from differences in death coding, data completeness, and other aspects of data comparability across countries.

## Key Points

**Question:**

What causes of death accounted for excess deaths in the US compared with other high-income countries from 1999 to 2022?

**Findings:**

In this cross-sectional study of more than 63.5 million deaths in the US, circulatory diseases accounted for the largest number of excess US deaths, increasing after 2001 for ages 45 to 64 years and after 2009 for ages 65 years or older. Drug poisonings, alcohol, and suicide accounted for 24% of the increase in excess US deaths overall and most of the increase for ages 0 to 44 years.

**Meaning:**

The findings suggest that the rise in excess mortality is due largely to increases in circulatory and metabolic diseases, drug poisonings, alcohol-related deaths, and suicide.

## Teaser

This cross-sectional study examines excess mortality in the US compared with other high-income countries from 1999 to 2022 and investigates causes of death accounting for the disparity.

## Introduction

US life expectancy has diverged from that of other high-income countries (HICs) since 1980, falling to 50th in global rankings in 2023.^1^ Between 1980 and 2021, an estimated 13 million US deaths could have been averted if US age-specific mortality rates had equaled those of other HICs.^2^

Quantifying the causes of death responsible for these “missing Americans”^2^ may help illuminate avenues for prevention. Prior studies of excess US deaths have focused on specific years,^3,4^ causes of death,^5,6,7^ age groups,^8,9^ or country comparisons.^10^ However, none offered a comprehensive analysis encompassing all leading causes of death by age and sex over the past 2 decades and the COVID-19 pandemic period. To address this gap, we performed a “population autopsy” of excess US deaths, drawing on cause-of-death data for all deaths occurring from 1999 to 2022 in the US and 17 other HICs.

## Methods

### Data Sources

In this cross-sectional study, we obtained raw death counts by underlying cause of death, sex, age, country, and year from the World Health Organization Mortality Database,^11^ which compiles data from national vital registration systems. Total resident population denominators were obtained from the Human Mortality Database.^12^ The study was determined not to be human participants research by the Boston University Medical Campus institutional review board and was thus exempted from approval and informed consent. Analyses were conducted from September 2023 to December 2025; data were downloaded regularly as the analysis proceeded, with the most recent download on June 3, 2025. The study followed the Strengthening the Reporting of Observational Studies in Epidemiology (STROBE) reporting guideline for cross-sectional studies.

### Inclusion Criteria

We included comparison HICs that had a 2021 gross domestic product per capita greater than US $24 000 and were not formerly part of the Soviet Union or Eastern bloc, for consistency with prior work.^2^ Our analysis started in 1999, which is when the *International Statistical Classification of Diseases and Related Health Problems, Tenth Revision (ICD-10)* was adopted. We excluded countries that lacked mortality data for 2020 through 2022 or failed to meet completeness and quality standards.^13^ Seventeen countries satisfied our inclusion criteria: Australia, Austria, Belgium, Canada, Denmark, Finland, France, Germany, Iceland, Italy, Japan, Luxembourg, the Netherlands, Spain, Sweden, Switzerland, and the United Kingdom (eTable 1 in Supplement 1). Data for 2021 and/or 2022 were imputed for 4 comparator countries (Germany, Belgium, Italy, and Japan) by applying proportional changes observed in the other comparator countries, stratified by age, sex, and cause (eTable 2 in Supplement 1).

### Cause-of-Death Classification

All deaths were assigned to an *ICD-10* code or to a residual (uncoded) category. We aggregated *ICD-10* codes into 17 mutually exclusive and collectively exhaustive cause-of-death categories, building on a classification by Elo et al.^14^ The categories, ranked by frequency, were circulatory diseases (eg, heart disease, hypertension, and stroke); other cancers (excluding lung cancer); mental and nervous system disorders (eg, Alzheimer disease and related dementias); respiratory diseases; lung cancer; diabetes, kidney, and metabolic diseases; infectious and parasitic diseases; influenza and pneumonia; drug poisonings; transportation accidents; COVID-19; symptoms, signs, and ill-defined conditions; suicide; alcohol-related mortality; homicide; HIV/AIDS; and all other causes. eTable 3 in Supplement 1 presents *ICD-10* codes for these cause-of-death categories.

### Statistical Analysis

We calculated cause-specific mortality rates for each age group (0-4, 5-14, 15-24, 25-34, 35-44, 45-54, 55-64, 65-74, 75-84, and ≥85 years), sex (male, female), country, and year by dividing cause-specific death counts by the total resident population. For each age group, sex, year, and cause-of-death category, we computed the population-weighted mean mortality rate in the 17 comparison HICs.

We quantified all-cause and cause-specific mortality gaps between the US and other HICs using 3 related but distinct metrics, stratified by age, sex, and year: (1) excess US deaths, quantified as the absolute difference between observed US deaths and deaths expected if US mortality rates were equal to the population-weighted mean rate of the other HICs; (2) excess years of life lost (YLL), calculated by multiplying excess US deaths by the number of additional years the deceased individuals would have been expected to live if they had survived^15^ (ie, the population-weighted mean life expectancy of a resident of the same age and sex and in the same year in other HICs); and (3) mortality rate ratios, computed by dividing the observed US death rates by the population-weighted mean of the rates of other HICs, with all rates standardized to the US population age distribution.

In each year from 1999 to 2022, we identified the contributions of different causes of death to the total number of excess US deaths and YLL. Specifically, we divided the number of excess US deaths and YLL attributable to each cause by the total number of excess US deaths and YLL in each year and multiplied by 100%. As there was no sampling, we do not present 95% CIs for these calculations.

For major causes of excess US deaths, we conducted linear joinpoint regression analyses on cause-specific excess deaths using the National Cancer Institute Joinpoint Regression program^16^ to assess the slopes and timing of trend breaks between 1999 and 2019, accounting for autocorrelated errors. To assess changes during the COVID-19 pandemic, we fit linear regression models to the 2014-2019 period and extrapolated the line through 2022 to assess deviations from prior trends.

All analyses were conducted using Stata/MP, version 18 (StataCorp LLC); R, version 4.5.1 (R Project for Statistical Computing); and Joinpoint, version 5.4.0 (National Cancer Institute). Analytic code is provided in the eAppendix in Supplement 1. Two-sided *P* < .05 was considered significant.

## Results

From 1999 to 2022, 63 547 318 deaths occurred in the US (49.6% among females and 50.4% among males; mean [SD] age at death, 73.2 [18.5] years), and 117 130 266 deaths occurred in the 17 other HICs; 63 179 958 of the US deaths (99%) and 115 356 631 of the peer country deaths (99%) had a valid underlying cause of death recorded for analysis (eTable 1 in Supplement 1). Over the 24-year period, the US experienced an estimated 12 675 646 more deaths—and 314 307 151 more YLL—than would have occurred if the US had death rates equal to other HICs (eTable 4 and eFigures 1 and 2 in Supplement 1). The annual number of excess US deaths increased steadily from 1999 to 2019 (1999: 346 166 deaths; 2009: 395 931 deaths; 2019: 636 306 deaths) and substantially during the COVID-19 pandemic (2020: 1 008 143 deaths; 2021: 1 174 286 deaths; 2022: 905 159 deaths) (Table and eTables 5-9 in Supplement 1). By the end of our study period in 2022, all-cause mortality rates were 1.38 times as high in the US as in other HICs.

**Table.** Excess Deaths and Excess YLL in the US Compared With Other High-Income Countries in 2022, by Cause of Death^a^

Cause of death	Deaths			Excess counts, No. (%)	
	Observed in US	Counterfactual^b^	Ratio of observed to counterfactual^b^	US deaths	US YLL^c^
All causes	3 279 754	2 374 595	1.38	905 159 (100)	21 884 041 (100)
Circulatory	925 392	566 819	1.63	358 573 (40)	5 920 216 (27)
Mental and nervous system disorders	386 728	207 612	1.86	179 116 (20)	1 963 129 (9)
Diabetes, kidney, and metabolic conditions	206 010	91 472	2.25	114 538 (13)	2 221 392 (10)
Drug poisoning	107 930	14 426	7.48	93 504 (10)	3 615 023 (17)
COVID-19	186 551	93 260	2.00	93 291 (10)	1 734 445 (8)
Respiratory	216 325	146 243	1.48	70 082 (8)	1 301 455 (6)
Transport accidents	49 175	12 009	4.09	37 166 (4)	1 430 404 (7)
Alcohol-related	51 186	24 192	2.12	26 994 (3)	837 510 (4)
Infectious or parasitic	64 995	38 536	1.69	26 459 (3)	612 908 (3)
Homicide	24 640	1729	14.25	22 911 (3)	1 079 635 (5)
Suicide	44 524	33 871	1.31	10 653 (1)	440 241 (2)
HIV/AIDS	4941	802	6.16	4139 (<1)	121 708 (1)
Lung cancer	131 981	131 463	1.00	518 (<1)	56 629 (<1)
Influenza or pneumonia	47 051	76 584	0.61	−29 533 (−3)	−124 476 (−1)
Other cancers^d^	492 467	531 519	0.93	−39 052 (−4)	−155 606 (−1)
Symptoms, signs, and ill-defined conditions	49 647	166 169	0.30	−116 522 (−13)	−1 211 173 (−6)
All other causes	290 211	237 889	1.22	52 322 (6)	2 040 600 (9)

Abbreviation: YLL, years of life lost.

^a^ Excess US deaths refer to the difference between observed US deaths and the numbers expected if US mortality rates were equal to the population-weighted mean rate of 17 other high-income countries (described in eTable 1 in Supplement 1). Excess YLL was calculated by multiplying excess US deaths by the number of additional years the deceased individuals would have been expected to live if they had survived.

^b^ Counterfactual refers to the expected number of deaths the US would have experienced if it had death rates equal to the population-weighted mean rate of the 17 other high-income countries.

^c^ YLL were summed across excess US deaths.

^d^ Includes all cancers other than lung.

### Causes of Excess US Deaths and YLL in 2022

The leading causes of excess US deaths in 2022 (n = 905 159) were circulatory diseases (358 573 deaths [40% of all excess deaths]); mental and nervous system disorders (179 116 [20%]); and diabetes, kidney, and metabolic conditions (114 538 [13%]). Excess deaths due to circulatory and metabolic conditions constituted 52% of all excess US deaths. An additional 131 151 excess US deaths (14% of total excess deaths) were due to drug poisonings (93 504 [10%]), alcohol (26 994 [3%]), and suicide (10 653 [1%]). Other major causes accounting for excess US deaths in 2022 were respiratory conditions (70 082 [8%]), transportation accidents (37 166 [4%]), and homicides (22 911 [3%]).

**Figure 1.**
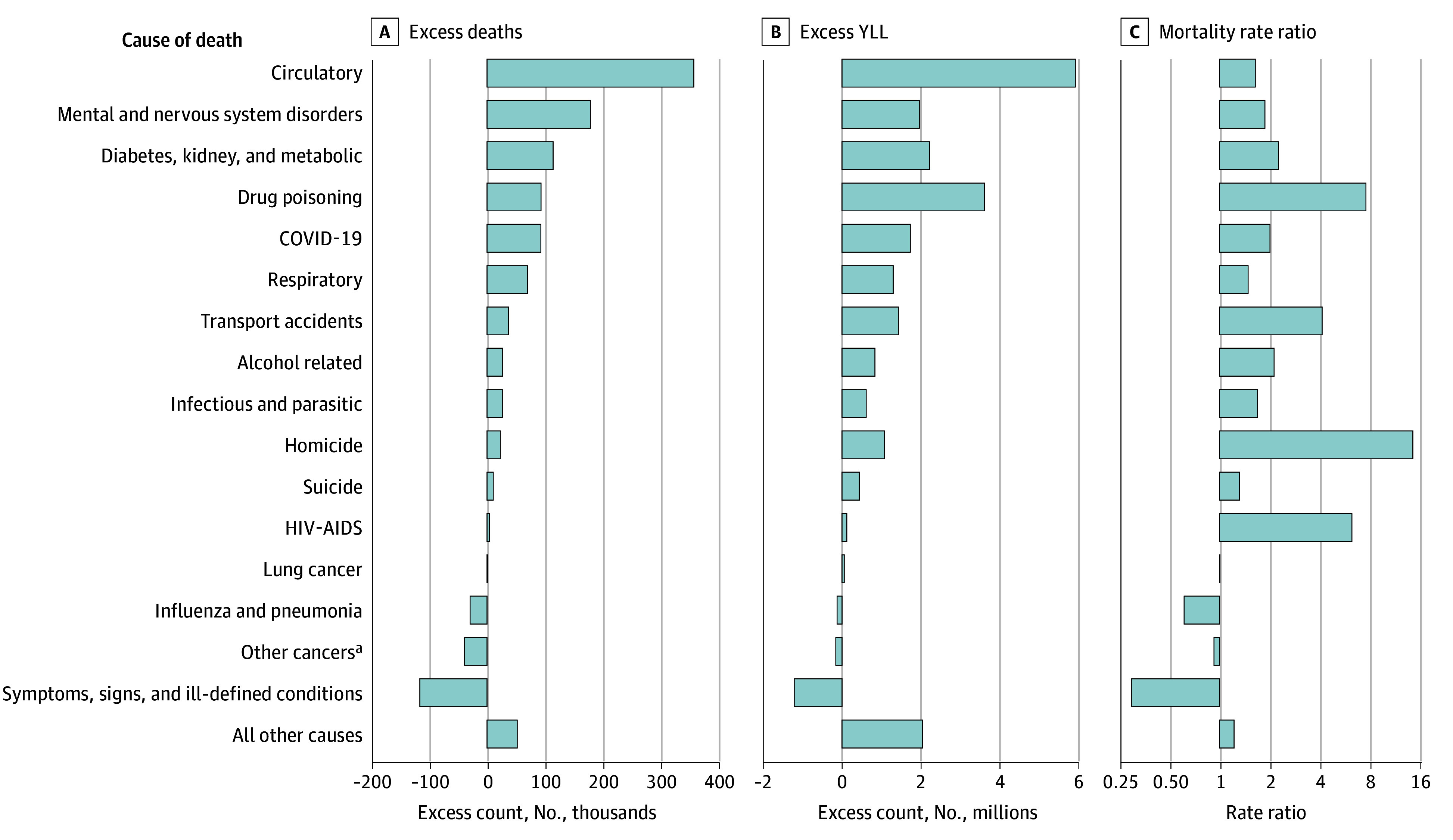
Bar Graphs Showing Excess Deaths and Excess Years of Life Lost (YLL) in the US Compared With Other High-Income Countries in 2022, by Cause of Death This figure is a visualization of data from the Table. The mortality rate ratio refers to the ratio of the age-standardized death rate for a cause of death in the US compared with the population-weighted mean rate of 17 other high-income countries. YLL were summed across excess US deaths. ^a^Other cancers include all cancers other than lung.

YLL is a measure that places greater weight on deaths earlier in life and thus offers a distinct perspective on the US excess mortality burden. In 2022, excess US deaths accounted for 21 884 041 excess US YLL (Figure 1 and Table). Circulatory diseases contributed less to excess US YLL than to excess US deaths (27% of YLL vs 40% of excess deaths), as did mental and nervous system disorders (9% of YLL vs 20% of excess deaths) and diabetes, kidney, and metabolic conditions (10% of YLL vs 13% of excess deaths). In contrast, drug poisonings (17% of YLL vs 10% of excess deaths), alcohol (4% of YLL vs 3% of excess deaths), and suicide (2% of YLL vs 1% of excess deaths) collectively contributed more to excess US YLL than to excess US deaths (22% of total YLL vs 14% of total excess deaths). Collectively, deaths due to drugs, alcohol, and suicide were the second leading cause of excess US YLL, totaling 4 892 774 in 2022.

The causes accounting for the largest number of excess US deaths in 2022 were not always the causes with the largest mortality rate ratios for the US compared with peer countries. Death rates for homicide and HIV/AIDS were 14.25 times and 6.16 times higher, respectively, in the US compared with peer countries (Table). Despite large relative differences, these causes accounted for just 3% and less than 1% of excess US deaths, respectively. On the other hand, circulatory death rates were 1.63 times as high in the US as in other HICs but were the leading cause of excess US deaths in 2022. In 2022, deaths from drug poisonings, accounting for 10% of excess deaths, were 7.48 times higher in the US than in peer countries. Deaths from diabetes, kidney, and metabolic conditions (13% of excess US deaths) were 2.25 times higher (Table).

### Changes in Causes of Excess US Deaths From 1999 to 2022

**Figure 2.**
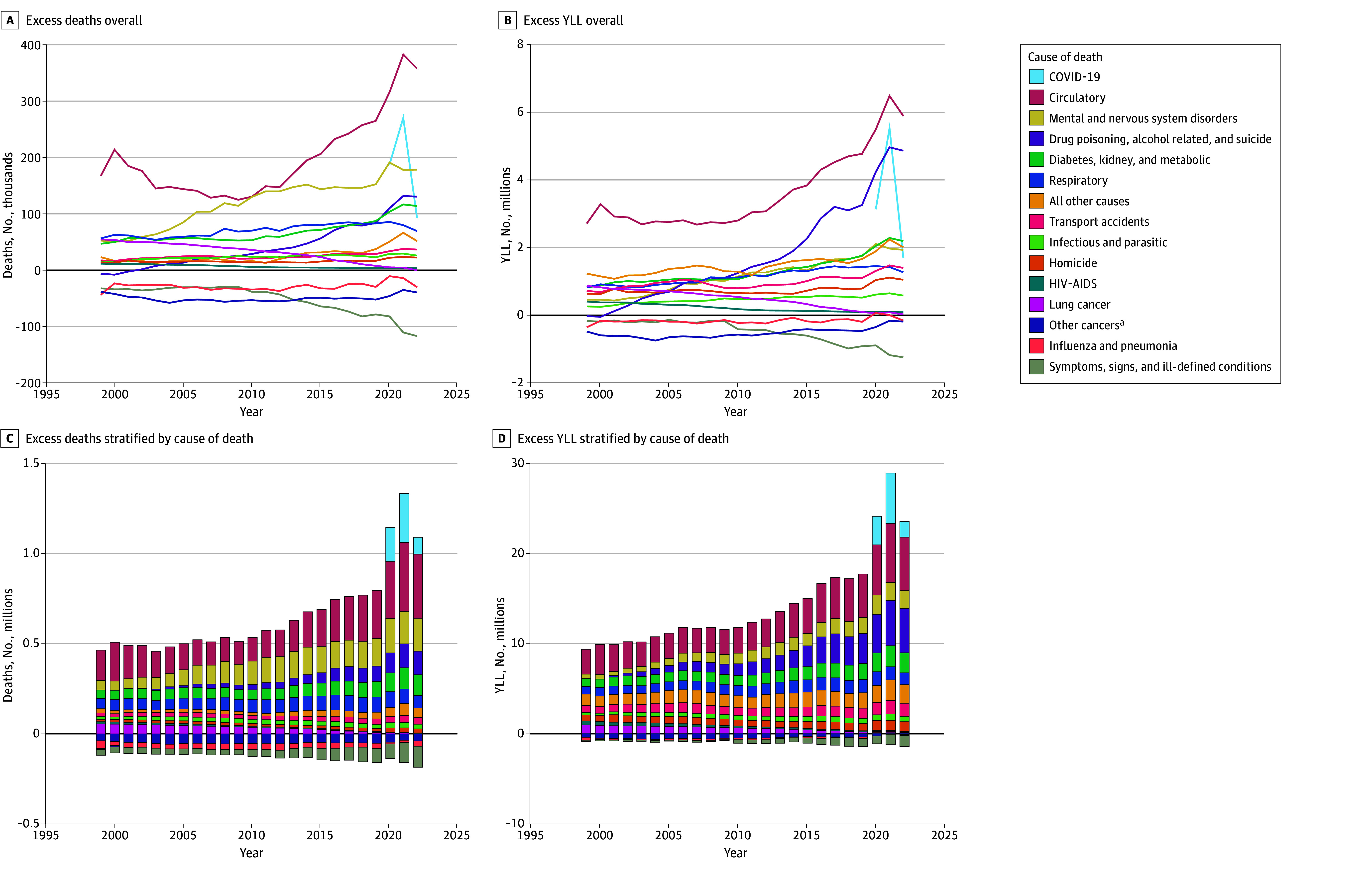
Line and Bar Graphs Showing Annual Excess Deaths and Excess Years of Life Lost (YLL) in the US Compared With Other High-Income Countries From 1999 to 2022, by Cause of Death C, D, Stacked bar graphs decompose excess US deaths and YLL by cause of death to demonstrate the contribution of each cause to the total excess. YLL were summed across excess US deaths. ^a^Other cancers include all cancers other than lung.

**Figure 3.**
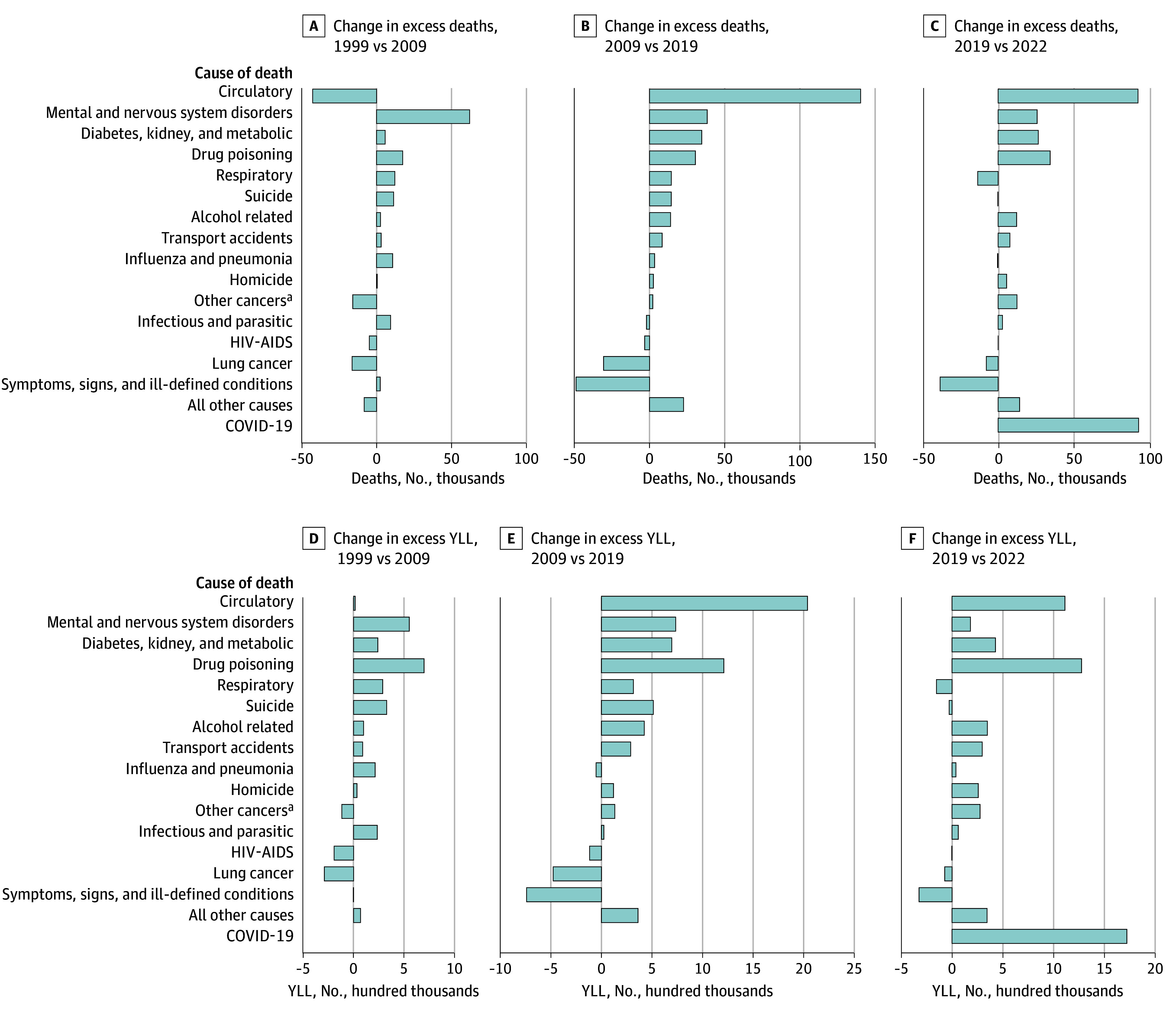
Bar Graphs Showing Changes in Excess US Deaths and Excess US Years of Life Lost (YLL) Over 3 Time Intervals Excess US deaths refer to the difference between observed US deaths and deaths expected if US mortality rates were equal to the population-weighted mean rate of 17 other high-income countries. YLL were summed across excess US deaths. ^a^Other cancers include all cancers other than lung.

Circulatory diseases were the leading cause of excess US deaths and YLL every year from 1999 to 2022 (except 2010) (Figure 2 and eFigure 3 in Supplement 1). From 1999 to 2009, the number of excess US deaths from circulatory diseases declined by 7809 (95% CI, 5505-10 112) per year. This trend reversed in 2009, as excess circulatory deaths increased by 15 474 (95% CI, 13 541-17 408) annually from 2009 to 2019 (eFigure 4 in Supplement 1). Between 2009 and 2022, circulatory diseases were the cause of death that contributed most to the increase in excess US deaths and excess US YLL (Figure 3).

Diabetes, kidney, and metabolic disease also became a more prominent cause of excess US deaths during the study period, rising from the fifth to the third leading cause (eFigure 3 in Supplement 1). Excess deaths from these conditions did not increase significantly from 1999 to 2010 (174 [95% CI, −416 to 766] excess deaths per year) but increased from 2010 to 2019 (3513 [95% CI, 2778-4247] excess deaths per year) (Figure 3 and eFigure 4 in Supplement 1).

Excess US deaths from mental and nervous system disorders also increased rapidly from 1999 (53 113 deaths) to 2009 (114 911 deaths), the largest increase of any cause during the first decade of the study period (Figure 2 and Figure 3). After 2013, excess US deaths due to mental and nervous system disorders plateaued. Nonetheless, there were more than 3 excess US deaths from mental and nervous system disorders at the end of the study period (179 116 deaths in 2022) for every 1 in 1999.

Excess US deaths from drug poisonings, alcohol, and suicide, collectively, rose steadily from 1999 (−5762 excess deaths) to 2022 (131 151 excess deaths), for a total increase of 136 912 annual excess US deaths (drug poisoning: 82 416 [15% of the total increase in excess deaths]; alcohol: 28 910 [5%]; suicide: 25 586 [5%]) (Figure 2 and eTable 10 in Supplement 1). Excess US deaths and YLL due to these causes increased particularly rapidly starting in 2013 (eFigure 4 in Supplement 1). Together, these causes accounted for 24% of the total increase in excess US deaths from 1999 to 2022 (eTable 10 in Supplement 1). This increase was even greater in terms of YLL (36% of the total increase).

COVID-19 was a major cause of excess US deaths from 2020 to 2022 (2020: 19%; 2021: 23%; 2022: 10%). The COVID-19 pandemic also coincided with sharp increases in excess US deaths for several other causes. Excess US deaths increased markedly in 2020 and did not return to prepandemic trends in 2021 or 2022 for circulatory diseases (358 573 excess deaths in 2022 vs 314 300 [95% CI, 295 000-333 600] anticipated excess deaths, based on trends from 2014 to 2019); mental and nervous system disorders (179 116 excess deaths vs 150 100 [95% CI, 135 300 to 165 000] anticipated deaths); and drug poisonings, alcohol, and suicide (131 151 excess deaths vs 111 000 [95% CI, 90 600 to 131 400] anticipated deaths) (eFigure 5 in Supplement 1).

### Causes of Excess US Deaths by Age and Sex

**Figure 4.**
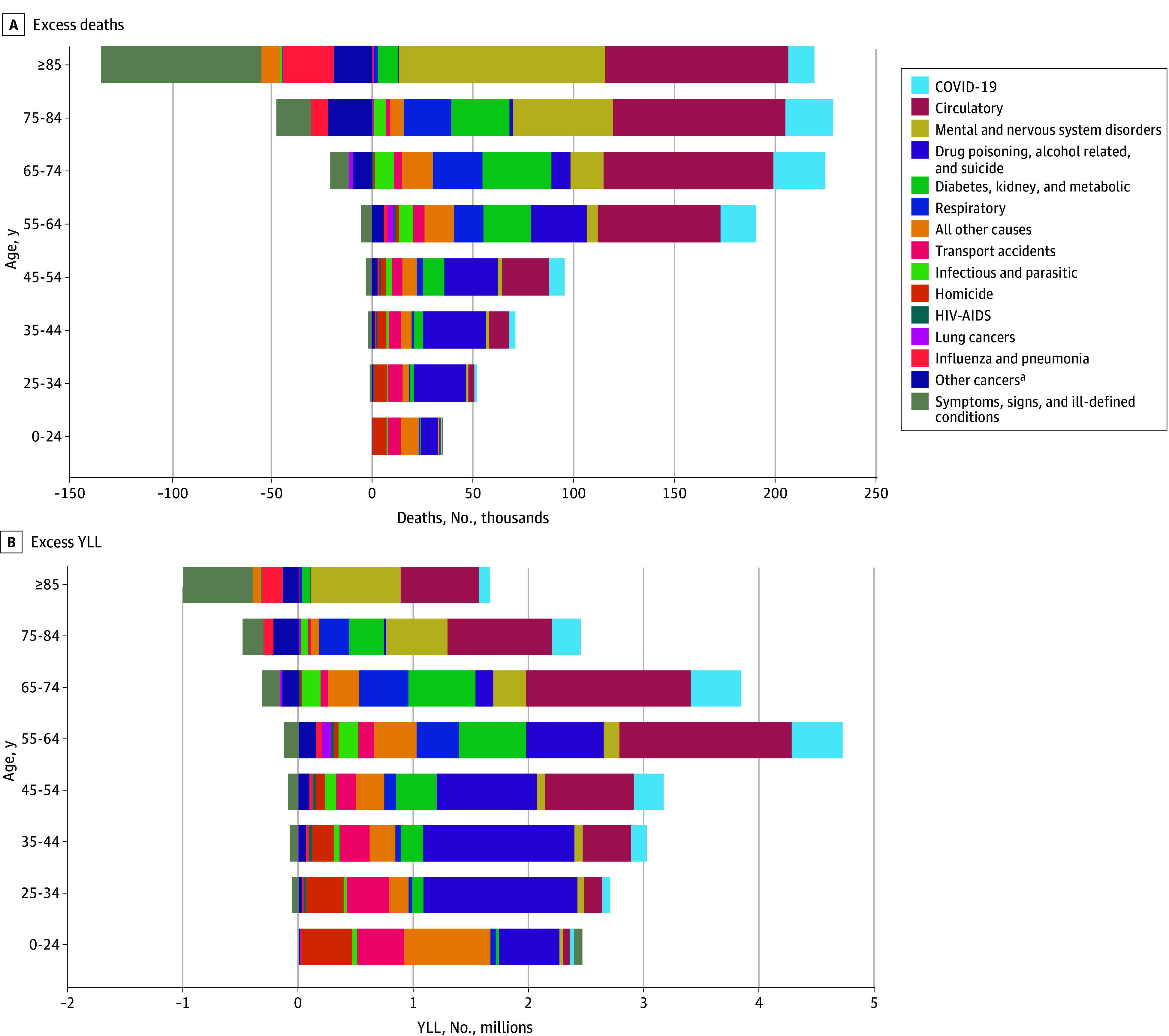
Bar Graphs Showing Age Distribution of Excess US Deaths and Excess US YLL in 2022, by Cause of Death Excess US deaths refer to the difference between observed US deaths and deaths expected if US mortality rates were equal to the population-weighted mean rate of 17 other high-income countries. YLL were summed across excess US deaths. ^a^Other cancers include all cancers other than lung.

Transportation accidents, homicides, drug poisonings, alcohol-related mortality, and suicide constituted most of the excess US deaths in 2022 that occurred among individuals aged 0 to 24 years (Figure 4). Drug poisonings, alcohol, and suicide accounted for nearly half of excess US deaths at ages 25 to 44 years and large numbers of excess US deaths at ages 45 to 54 and 55 to 64 years (eTable 11 in Supplement 1). Circulatory diseases and metabolic conditions together accounted for nearly half of excess US deaths for persons aged 55 to 64 years and more than half of excess US deaths for ages 65 to 74 and 75 to 84 years. Mental and nervous system disorders were the leading cause of excess US deaths in people aged 85 years or older. There were fewer deaths attributed to symptoms, signs, and ill-defined conditions in the US compared with other HICs, particularly for people aged 85 years or older.

The rise in excess US deaths at age 0 to 44 years over the study period was due predominately to increases in drug poisonings, alcohol-related mortality, and suicide (eFigures 6 and 7 in Supplement 1). For persons aged 45 to 64 years, the rise in excess US deaths was driven by (1) a steep rise in excess mortality due to drug poisonings, alcohol, and suicide; (2) a steady rise in excess mortality due to circulatory diseases and to diabetes, kidney, and metabolic conditions; and (3) COVID-19 from 2020 to 2022. For persons aged 65 years or older, the rise in excess US deaths was driven in the first decade (1999 to 2009) by an increase in mental and nervous system conditions, in the second decade (2010 to 2019) by an increase in circulatory mortality, and from 2020 to 2022 by COVID-19. Excess US deaths from circulatory diseases exhibited different trends at ages 45 to 64 years than ages 65 years or older. For ages 45 to 64 years, excess US circulatory deaths began to rise in 2001, whereas at ages 65 years or older, excess US circulatory deaths decreased from 1999 to 2009 and then rose steeply after 2009.

Across the study period, mental and nervous system disorders and lung cancer contributed more to excess US deaths among females, whereas homicide and transport accidents contributed more to excess US deaths among males (eFigure 8 and eTable 12 in Supplement 1). Excess US deaths due to drug poisoning, alcohol, and suicide also made up a larger share of excess US deaths for males than females.

## Discussion

In this study, we conducted a population autopsy to quantify the burden and causes of excess US deaths—deaths that would have been avoided had US mortality rates equaled the population-weighted mean rate of the 17 other HICs. We found that annual excess US deaths increased from 346 166 in 1999 to 905 159 in 2022, during the COVID-19 pandemic. Circulatory diseases were the leading cause of excess US deaths nearly every year, and although annual rates of excess circulatory disease deaths decreased from 1999 to 2009, they increased markedly from 2009 to 2022. Excess US deaths from diabetes, kidney, and metabolic disease followed a similar pattern, with no significant increase in annual rates from 1999 to 2010 but with rates rapidly increasing from 2010 to 2022. Drug poisonings, alcohol-related mortality, and suicide were also a major cause of the increase in excess US deaths, especially among men. Together, these causes accounted for 24% of the increase in excess US deaths overall from 1999 to 2022 and most of the increase in excess US deaths for individuals aged 0 to 44 years. At ages 85 years or older, mental and nervous system disorders (eg, Alzheimer disease and related dementias) were a large and growing cause of excess US deaths. The pandemic also brought additional divergence of US mortality from peer nations. While COVID-19 accounted for 19% of excess US deaths in 2020, excess US deaths from circulatory disease; diabetes, kidney, and metabolic disease; and drug poisonings, alcohol-related mortality, and suicide also increased markedly throughout the pandemic.

The most prevalent causes of excess US deaths represent the greatest opportunity to reduce the US mortality gap relative to other HICs. In 2022, US death rates from circulatory and metabolic diseases were 1.63 and 2.25 times as high as in other HICs, respectively, and together these conditions accounted for over half of excess US deaths. While circulatory disease mortality has steadily declined in other HICs, it plateaued in the US after 2009. Mehta et al^7^ estimated that continued declines in US cardiovascular mortality during the 2010s would have closed the life expectancy gap with peer nations. Our findings add nuance to this narrative, highlighting age-specific trends. The rise in excess circulatory disease mortality among adults aged 45 to 64 years began a full decade earlier than among older adults, suggesting that cohort-specific exposures, such as higher obesity prevalence,^17^ may play an important role in these trends. Deaths due to diabetes, kidney, and metabolic conditions showed similar patterns, consistent with evidence of elevated cardiometabolic risk factors, including obesity, hyperglycemia, and poor kidney function, in the US compared with other HICs.^18,19,20^

Drug poisonings, alcohol-related causes, and suicide—commonly referred to as deaths of despair^10,21,22,23,24^—accounted for the most rapid increase in excess US deaths, rising from near parity with peer countries in 1999 to more than 130 000 excess US deaths in 2022, with a particularly rapid rise occurring after 2013 once fentanyl was widely introduced into US drug supplies. Drug poisonings, alcohol, and suicide resulted in more YLL than any other cause except circulatory diseases, and increases in these causes of death accounted for most of the US mortality divergence from other HICs at ages 0 to 44 years and a substantial part of the divergence at ages 45 to 64 years. Consistent with the literature on deaths of despair that has documented distinct sex patterns in mortality trends,^21,22^ this study found that excess US deaths from drug poisonings, alcohol, and suicide were more common among males. The outsized contribution of drug poisonings, alcohol, and suicide to excess US YLL compared with excess US deaths highlights how these deaths tend to occur at younger ages. Given that deaths of despair are closely associated with social disadvantage,^10,21,22,23,24^ premature mortality from drug poisonings, alcohol, and suicide may represent a key pathway through which US health, social, and economic policy has led to mortality divergence from other HICs.

Overall, these findings reveal a 2-part narrative behind the growing mortality disadvantage^25,26,27^ in the US compared with peer countries leading up to the COVID-19 pandemic: (1) a rapid increase in deaths from drug poisoning, alcohol-related causes, and suicide, occurring predominately at younger ages, and (2) a steady rise in deaths from circulatory and metabolic disease, occurring mostly in midlife and among older adults. The COVID-19 pandemic also contributed to a sharp rise in excess US deaths in 2020 and 2021. Excess COVID-19 deaths represented approximately 1 in 5 excess US deaths in 2020 and 2021. Excess deaths from other causes were also amplified during the pandemic, including circulatory diseases, metabolic conditions, drug poisonings, alcohol, and suicide—likely reflecting disruptions to care, behavioral health, and social determinants of health as well as misattribution of some COVID-19–related deaths to other causes. The effects of the pandemic were attenuated in 2022, and prior research shows that by 2023, the number of excess US deaths matched the prepandemic trend.^28^

Excess US deaths may be avoidable with existing policy interventions available in other HICs. In this way, international comparisons of mortality are useful because they reveal health gaps attributable to national policy environments that are not visible when comparing population groups within a country or over time. Furthermore, comparing US mortality rates with those of other HICs may offer more achievable and policy-relevant estimates of avoidable deaths than classifying all deaths due to prespecified causes as “preventable,” “treatable,” or “medically amenable,” as in prior studies.^29^

While our population autopsy quantified the role of different proximate causes of death in the US mortality disadvantage, these patterns reflect cross-national differences in social, economic, environmental, political, and policy factors.^30,31,32,33,34^ Several studies have associated opioid overdoses and cardiovascular mortality in the US with social and economic changes, including trade liberalization, the loss of manufacturing jobs, automation, and worsened opportunities for less-educated workers.^34,35,36,37^ US regions with less-protective safety net and health coverage policies have higher and worsening mortality than other regions,^38,39,40^ as do those where majorities voted for Donald Trump in 2016.^40,41^ Other studies have explored supply-side causes of mortality trends,^42^ including the overprescription of opioids starting in the late 1990s,^43^ the proliferation of fentanyl since 2013,^5^ and the rise of obesity and consequent metabolic disease associated with changes in the food environment.^19^ Our findings underscore the need for further research into several critical areas: reasons for the accelerating US mortality divergence after 2010 across multiple causes of death, differences in US cardiometabolic mortality trends by age group, the rapid increase in drug poisoning deaths among young adults, and the rise in mental and nervous system deaths among older adults.

### Limitations

Our study has several limitations. First, these descriptive findings should be interpreted in light of several different sources of uncertainty: stochastic variation in annual death rates, measurement error from differences in diagnostic practices, coding practices, data completeness, and pandemic-era reporting between countries and over time, as well as assumptions involved in the imputation and extrapolation of missing data. Second, we followed prior practice in choosing all available comparator countries to estimate excess US deaths.^2^ As more countries report cause-disaggregated data, the addition of other countries may yield somewhat different estimates. Third, peer countries had higher death rates attributed to symptoms, signs, and ill-defined conditions, particularly later in the study period. This may reflect reporting lags which, when resolved, will increase deaths attributed to other categories. Fourth, death certificates do not assign causality to important upstream determinants of mortality, such as poverty, social disadvantage, racism, and other structural determinants of health, which are likely to be critical targets for intervention.^44^ Fifth, while we stratified by age and sex, our analysis did not assess differences by race and ethnicity, educational attainment, or geography. Sixth, data on cause-specific mortality were available only through 2022 for this analysis. Recent evidence indicates that excess US deaths from all causes continued to rise in 2023, even after the acute phase of the COVID-19 pandemic.^45^

## Conclusions

In this repeated cross-sectional study of cross-national mortality, we provided a comprehensive assessment of the causes of excess US deaths compared with other HICs over 24 years. Increases in circulatory and metabolic diseases as well as drug poisonings, alcohol-related deaths, and suicide contributed the most to excess deaths and YLLs. Despite similar access to advanced medical technology, the US has experienced persistently higher death rates than other HICs, possibly driven by differences in health, economic, and social policy.

## Notes

Supplement 1. **eTable 1.** Total Deaths and Person-Time in the US and 17 Other High-Income Countries by Cause of Death From 1999 to 2022 **eTable 2.** Availability of Cause-Specific Mortality Data by Country as of June 2025 **eTable 3.** *ICD-10* Code Typology for Cause of Death Categories Used in This Analysis **eTable 4.** Excess Deaths and Excess YLL in the US Compared With Other High-Income Countries by Cause of Death From 1999 to 2022 **eTable 5.** Excess Deaths and Excess YLL in the US Compared With Other High-Income Countries by Cause of Death in 1999 **eTable 6.** Excess Deaths and Excess YLL in the US Compared With Other High-Income Countries by Cause of Death in 2009 **eTable 7.** Excess Deaths and Excess YLL in the US Compared With Other High-Income Countries by Cause of Death in 2019 **eTable 8.** Excess Deaths and Excess YLL in the US Compared With Other High-Income Countries by Cause of Death in 2020 **eTable 9.** Excess Deaths and Excess YLL in the US Compared With Other High-Income Countries by Cause of Death in 2021 **eTable 10.** Changes in Excess US Deaths and Excess US YLL by Cause of Death From 1999 to 2019, 2019 to 2022, and 1999 to 2022 **eTable 11.** Age Distribution of Excess US Deaths and Excess US YLL by Cause of Death in 2022 **eTable 12.** Excess US Deaths and Excess US YLL by Sex and Cause of Death From 1999 to 2022 **eFigure 1.** Annual Observed Deaths in the US, Counterfactual Deaths in Other High-Income Countries, and Excess Deaths in the US Compared With Other High-Income Countries for Each Cause of Death From 1999 to 2022 **eFigure 2.** Annual Observed YLL in the US, Counterfactual YLL in Other High-Income Countries, and Excess YLL in the US Compared With Other High-Income Countries for Each Cause of Death From 1999 to 2022 **eFigure 3.** Rank Order of Excess US Deaths by Cause From 1999 to 2022 **eFigure 4.** Change in Slope Across Time Periods for Top 4 Causes of Excess US Deaths **eFigure 5.** Regression Analysis of Prepandemic Trends of Top 4 Causes of Excess US Deaths **eFigure 6.** Annual Excess US Deaths by Age Group and Cause of Death From 1999 to 2022 **eFigure 7.** Annual Excess US Deaths and Excess US YLL by Age Group and Cause of Death From 1999 to 2022 **eFigure 8.** Annual Excess US Deaths and Excess US YLL by Sex and Cause of Death From 1999 to 2022 **eAppendix.** Supplementary Data and Replication Code

Supplement 2.
Data Sharing Statement
