# Supplementary material for: Causes of Excess Deaths in the US Compared With Other High-Income Countries
Source: JAMA Netw Open. 2026 May 8;9(5):e266147. doi: 10.1001/jamanetworkopen.2026.6147 (PMC13156795; doi:10.1001/jamanetworkopen.2026.6147)
Supplement: Supplement 1. — eTable 1. Total Deaths and Person-Time in the US and 17 Other High-Income Countries by Cause of Death From 1999 to 2022 eTable 2. Availability of Cause-Specific Mortality Data by Country as of June 2025 eTable 3. ICD-10 Code Typology for Cause of Death Categories Used in This Analysis eTable 4. Excess Deaths and Excess YLL in the US Compared With Other High-Income Countries by Cause of Death From 1999 to 2022 eTable 5. Excess Deaths and Excess YLL in the US Compared With Other High-Income Countries by Cause of Death in 1999 eTable 6. Excess Deaths and Excess YLL in the US Compared With Other High-Income Countries by Cause of Death in 2009 eTable 7. Excess Deaths and Excess YLL in the US Compared With Other High-Income Countries by Cause of Death in 2019 eTable 8. Excess Deaths and Excess YLL in the US Compared With Other High-Income Countries by Cause of Death in 2020 eTable 9. Excess Deaths and Excess YLL in the US Compared With Other High-Income Countries by Cause of Death in 2021 eTable 10. Changes in Excess US Deaths and Excess US YLL by Cause of Death From 1999 to 2019, 2019 to 2022, and 1999 to 2022 eTable 11. Age Distribution of Excess US Deaths and Excess US YLL by Cause of Death in 2022 eTable 12. Excess US Deaths and Excess US YLL by Sex and Cause of Death From 1999 to 2022 eFigure 1. Annual Observed Deaths in the US, Counterfactual Deaths in Other High-Income Countries, and Excess Deaths in the US Compared With Other High-Income Countries for Each Cause of Death From 1999 to 2022 eFigure 2. Annual Observed YLL in the US, Counterfactual YLL in Other High-Income Countries, and Excess YLL in the US Compared With Other High-Income Countries for Each Cause of Death From 1999 to 2022 eFigure 3. Rank Order of Excess US Deaths by Cause From 1999 to 2022 eFigure 4. Change in Slope Across Time Periods for Top 4 Causes of Excess US Deaths eFigure 5. Regression Analysis of Prepandemic Trends of Top 4 Causes of Excess US Deaths eFigure 6. Annual Excess US Deaths by Ag [file jamanetwopen-e266147-s001.pdf]

Supplementary Online Content

Bor J, Raquib RV, Himmelstein D, Woolhandler S, Stokes AC. Causes of excess deaths in the US compared with other high-income countries. *JAMA Netw Open*. 2026;9(5):e266147. doi:10.1001/jamanetworkopen.2026.6147

eTable 1. Total Deaths and Person-Time in the US and 17 Other High-Income Countries by Cause of Death From 1999 to 2022

eTable 2. Availability of Cause-Specific Mortality Data by Country as of June 2025

eTable 3. *ICD-10* Code Typology for Cause of Death Categories Used in This Analysis

eTable 4. Excess Deaths and Excess YLL in the US Compared With Other High-Income Countries by Cause of Death From 1999 to 2022

eTable 5. Excess Deaths and Excess YLL in the US Compared With Other High-Income Countries by Cause of Death in 1999

eTable 6. Excess Deaths and Excess YLL in the US Compared With Other High-Income Countries by Cause of Death in 2009

eTable 7. Excess Deaths and Excess YLL in the US Compared With Other High-Income Countries by Cause of Death in 2019

eTable 8. Excess Deaths and Excess YLL in the US Compared With Other High-Income Countries by Cause of Death in 2020

eTable 9. Excess Deaths and Excess YLL in the US Compared With Other High-Income Countries by Cause of Death in 2021

eTable 10. Changes in Excess US Deaths and Excess US YLL by Cause of Death From 1999 to 2019, 2019 to 2022, and 1999 to 2022

eTable 11. Age Distribution of Excess US Deaths and Excess US YLL by Cause of Death in 2022

eTable 12. Excess US Deaths and Excess US YLL by Sex and Cause of Death From 1999 to 2022

eFigure 1. Annual Observed Deaths in the US, Counterfactual Deaths in Other High-Income Countries, and Excess Deaths in the US Compared With Other High-Income Countries for Each Cause of Death From 1999 to 2022

eFigure 2. Annual Observed YLL in the US, Counterfactual YLL in Other High-Income Countries, and Excess YLL in the US Compared With Other High-Income Countries for Each Cause of Death From 1999 to 2022

eFigure 3. Rank Order of Excess US Deaths by Cause From 1999 to 2022

eFigure 4. Change in Slope Across Time Periods for Top 4 Causes of Excess US Deaths

eFigure 5. Regression Analysis of Prepandemic Trends of Top 4 Causes of Excess US Deaths

eFigure 6. Annual Excess US Deaths by Age Group and Cause of Death From 1999 to 2022

© 2026 Bor J et al. *JAMA Network Open*.

eFigure 7. Annual Excess US Deaths and Excess US YLL by Age Group and Cause of Death From 1999 to 2022

eFigure 8. Annual Excess US Deaths and Excess US YLL by Sex and Cause of Death From 1999 to 2022

eAppendix. Supplementary Data and Replication Code

This supplementary material has been provided by the authors to give readers additional information about their work.

© 2026 Bor J et al. *JAMA Network Open*.

eTable 1. Total deaths and person-time in the US and 17 other high-income countries by cause of death from 1999 to 2022

	USA	Australia	Austria	Belgium	Canada	Switzerland	Denmark	Spain	Finland	France	Germany	Iceland	Italy	Japan	Luxembourg	Netherlands	Sweden	UK
Total																		
Total Deaths	63 547 318	3 407 411	1 686 363	2 457 010	5 857 645	1 551 461	1 316 708	9 604 675	1 239 136	12 923 652	19 196 750	49 359	11 636 206	27 229 702	90 416	3 470 837	2 211 215	13 201 720
Total Person-Time	4 168 540 637	233 164 944	117 070 002	181 715 190	389 122 796	110 949 099	92 249 423	681 650 482	84 391 597	898 003 593	1 484 047 505	3 430 886	873 462 599	2 038 296 165	6 252 890	241 660 377	161 361 912	922 632 565
Valid COD	63 179 958	3 389 707	1 663 545	2 412 121	5 785 336	1 501 415	1 252 746	9 485 589	1 231 213	12 429 889	18 770 849	48 933	11 571 706	27 004 627	89 558	3 373 622	2 176 101	13 169 674
% of total																		
Circulatory	32.4	31.0	41.3	30.2	28.0	34.0	27.6	30.5	38.7	25.7	41.3	34.3	36.7	28.1	33.7	28.3	38.5	30.0
Other cancers	16.6	24.0	21.1	20.1	21.9	21.3	22.3	21.5	18.9	23.3	20.9	22.0	23.1	24.4	21.6	23.1	21.1	21.8
Mental and nervous system disorders	9.8	9.5	4.9	7.8	10.6	10.9	7.7	8.9	16.3	8.8	5.3	12.0	6.8	3.0	7.5	9.8	9.7	10.4
Respiratory	7.3	6.7	4.1	6.9	6.3	4.1	7.3	8.7	3.1	4.3	4.6	5.2	5.5	5.9	5.6	5.7	4.1	8.0
Lung cancers	5.7	5.4	4.6	5.8	7.4	4.7	6.5	5.1	4.1	5.2	4.9	6.2	5.4	5.7	5.5	6.8	3.8	5.8
Diabetes, renal, and metabolic	5.7	5.4	6.3	3.5	5.0	3.3	3.8	4.7	1.5	3.9	4.7	2.6	5.6	3.4	3.3	3.7	3.3	2.0
Infectious & parasitic	2.2	1.5	0.9	2.1	1.6	1.0	1.6	1.4	0.6	1.8	1.6	0.9	1.7	2.0	2.0	1.6	1.9	1.1
Influenza and pneumonia	2.1	1.9	1.5	3.8	2.3	2.3	3.2	2.3	1.7	2.2	2.3	3.3	1.7	8.9	2.5	3.4	2.5	5.1
Drug poisoning	1.8	1.0	0.3	0.4	1.3	0.5	0.6	0.2	0.9	0.4	0.2	1.2	0.1	0.1	0.6	0.3	0.8	0.6
Transport accidents	1.7	1.1	0.7	0.9	1.0	0.6	0.6	0.9	0.8	0.7	0.6	0.9	0.7	0.7	0.9	0.6	0.5	0.4
COVID-19	1.5	0.3	1.2	1.3	0.9	1.2	0.3	1.5	0.5	1.3	0.2	0.5	1.2	0.1	1.2	1.4	0.8	1.4
Suicide	1.3	1.6	1.5	1.6	1.3	1.5	0.9	0.8	1.4	1.5	1.0	1.4	0.6	2.2	1.3	1.0	1.0	0.7
Alcohol-related	1.1	0.8	1.5	1.1	1.1	1.2	2.6	0.4	2.9	1.4	1.7	0.6	0.2	0.4	1.6	0.6	0.9	1.2
Homicide	0.7	0.2	0.1	0.1	0.2	0.1	0.1	0.1	0.2	0.1	0.1	0.1	0.1	0.0	0.1	0.1	0.1	0.0
HIV/AIDS	0.3	0.1	0.1	0.0	0.1	0.1	0.0	0.2	0.0	0.1	0.0	0.0	0.1	0.0	0.1	0.0	0.0	0.0
Symptoms, signs, and ill-defined conditions	1.4	0.9	2.4	4.6	1.7	4.2	6.7	2.5	0.7	8.5	2.9	1.1	2.1	6.4	3.3	4.7	3.3	2.2
All other causes	8.4	8.9	7.7	9.6	9.3	9.0	8.1	10.1	7.9	10.7	7.7	7.5	8.3	8.9	9.3	9.0	7.8	9.4

Abbreviations: COD = Cause of death. USA = United States. UK = United Kingdom

© 2026 Bor J et al. *JAMA Network Open*.

eTable 2. Availability of cause-specific mortality data by country as of June 2025

Country Code	Country	Years Available	Method
2450	United States	1999-2022	
4300	Switzerland	1999-2022	
4050	Denmark	1999-2022	
4280	Spain	1999-2022	
4070	Finland	1999-2022	
4160	Iceland	1999-2022	
4190	Luxembourg	1999-2022	
4210	Netherlands	1999-2022	
4290	Sweden	1999-2022	
2090	Canada	2000-2022	1999 excluded
4080	France	2000-2022	1999 excluded
4308	United Kingdom	2001-2022	1999-2000 excluded
4010	Austria	2002-2022	1999-2001 excluded
5020	Australia	1999-2004, 2006-2022	2005 linear interpolation between adjacent years within age-by-sex-by-cause strata
4020	Belgium	1999-2021	2022 imputed by applying OWN (excluding Germany, Belgium, Italy and Japan) proportional change to 2021 data
4180	Italy	2003-2021	1999-2002 excluded. 2022 imputed by applying OWN (excluding Germany, Belgium, Italy and Japan) proportional change to 2021 data
3160	Japan	1999-2021	2022 imputed by applying OWN (excluding Germany, Belgium, Italy and Japan) proportional change to 2021 data
4085	Germany	1999-2020	2021-2022 imputed by applying OWN (excluding Germany, Belgium, Italy and Japan) proportional change to 2020 data

Notes: Source - https://www.who.int/data/data-collection-tools/who-mortality-database

© 2026 Bor J et al. *JAMA Network Open*.

eTable 3. *ICD-10* code typology for cause of death categories used in this analysis

Categories	ICD-10 Codes
Circulatory diseases	I00–I99 (excluding I42.6)
Other cancers (excluding lung cancers)	C00–D48 (excluding C33, C34)
Mental and nervous system disorders[a]	F01–F99 (excluding F10), G00–G98 (excluding G31.2, G62.1, G72.1)
Respiratory diseases (excluding influenza and pneumonia)	J00–J98 (excluding J09–J18)
Lung cancers	C33–C34
Diabetes, renal, and metabolic diseases	E10–E14, N17–N19, E70–E88, E65–E68
Infectious and parasitic diseases	A00–B99 (excluding B20–B24)
Influenza and pneumonia	J09–J18
Transport accidents	V01–V99, Y85
Drug poisoning[b]	X40–X44, X60–X64, X85, Y10–Y14
Suicide	X66–X84, Y87.0
Alcohol-related causes	E24.4, F10, G31.2, G62.1 G72.1, I42.6, K29.2, K70, K85.2, K86.0, R78.0, X45, X65, Y15
Homicide[c]	X86–Y09, Y87.1
COVID-19	U07.1
HIV/AIDS	B20–B24
Symptoms, signs, and ill-defined conditions	R00–R99 (excluding R78.0)
All other causes	D50–D89, E00–E90 (excluding E10–E14, E65–E68, E70–E88), G99, H00–H93, J99, K00–K92, L00–L98, M00–M99, N00–N99 (excluding N17–N19), O00–O99, P00–P96, Q00–Q99, U00–U99 (excluding U07.1), V01–Y89 (excluding X40–X44, X45, X60–Y15, Y85, Y87.0–Y87.1), Y90–Y98

[a]Includes Alzheimer's disease and related dementias (ADRD).
[b]Except suicide by drugs, which is included in drug overdose.
[c]Except assault by drugs, medicaments, and biological substances, which is included in drug overdose.
Notes: This list is ordered by the number of deaths that occurred in the US from 1999 to 2022, besides symptoms, signs and ill-defined conditions, and all other causes. To modify the list from Elo et al., we added the following cause-of-death categories: transport accidents, COVID-19, and infectious and parasitic disease. We expanded the breast, prostate, colorectal, and cervical cancer category to include all other cancers besides lung cancers, and the diabetes category to include renal and metabolic diseases.

© 2026 Bor J et al. *JAMA Network Open*.

eTable 4. Excess deaths and excess YLL in the US compared with other high-income countries by cause of death from 1999 to 2022

				Excess US deaths		Excess US YLL	
Causes of death	Observed US deaths	Counterfactual deaths	Ratio of observed to counterfactual	No.	% of total	No.	% of total
All-cause	63 547 318	50 871 672	1.25	12 675 646	100	314 307 151	100
Circulatory	20 596 765	15 821 002	1.30	4 775 763	38	87 712 574	28
Mental and nervous system disorders	6 213 197	3 329 800	1.87	2 883 397	23	28 855 758	9
Respiratory	4 626 079	2 915 668	1.59	1 710 411	13	28 195 135	9
Diabetes, renal, and metabolic	3 633 773	1 985 778	1.83	1 647 995	13	32 318 223	10
Drug poisoning	1 146 815	224 389	5.11	922 426	7	36 445 349	12
Lung cancers	3 649 100	2 880 915	1.27	768 185	6	12 164 914	4
Transport accidents	1 055 959	445 034	2.37	610 925	5	24 377 753	8
Infectious & parasitic	1 387 055	820 131	1.69	566 924	4	11 938 532	4
COVID-19	954 268	401 509	2.38	552 759	4	10 458 675	3
Homicide	445 248	48 489	9.18	396 759	3	18 769 275	6
HIV/AIDS	220 660	44 761	4.93	175 899	1	5 883 794	2
Alcohol-related	711 267	576 993	1.23	134 274	1	4 991 957	2
Suicide	824 259	828 737	0.99	-4 478	0	2 117 039	1
Influenza and pneumonia	1 346 039	2 026 488	0.66	-680 449	-5	-3 214 541	-1
Other cancers	10 565 935	11 748 441	0.90	-1 182 506	-9	-11 735 684	-4
Symptoms, signs, and ill-defined conditions	859 990	2 103 795	0.41	-1 243 805	-10	-11 011 260	-4
All other causes	5 310 909	4 669 742	1.14	641 167	5	36 039 657	11

Abbreviations: YLL = Years of life lost (summed across excess US deaths)
Notes: Notes: The counterfactual refers to the expected number of deaths the US would have experienced if it had death rates equal to the average of 17 other high-income countries, which are described in eTable 1.

© 2026 Bor J et al. *JAMA Network Open*.

eTable 5. Excess deaths and excess YLL in the US compared with other high-income countries by cause of death in 1999

				Excess US deaths		Excess US YLL	
Causes of death	Observed US deaths	Counterfactual deaths	Ratio of observed to counterfactual	No.	% of total	No.	% of total
All-cause	2 391 043	2 044 877	1.17	346 166	100	8 341 146	100
Circulatory	953 720	785 731	1.21	167 989	49	2 740 793	33
Respiratory	166 129	109 193	1.52	56 936	16	851 171	10
Lung cancers	152 154	97 066	1.57	55 088	16	898 424	11
Mental and nervous system disorders	119 932	66 819	1.79	53 113	15	490 702	6
Diabetes, renal, and metabolic	120 589	73 136	1.65	47 453	14	853 438	10
Transport accidents	46 386	28 481	1.63	17 905	5	751 493	9
Infectious & parasitic	45 181	29 062	1.55	16 119	5	292 491	4
Homicide	16 800	2 543	6.61	14 257	4	667 411	8
HIV/AIDS	14 801	2 617	5.66	12 184	4	436 618	5
Drug poisoning	16 833	5 745	2.93	11 088	3	417 030	5
Alcohol-related	19 461	21 377	0.91	-1 916	-1	-35 547	0
Suicide	25 979	40 913	0.63	-14 934	-4	-373 282	-4
Symptoms, signs, and ill-defined conditions	26 597	58 245	0.46	-31 648	-9	-137 327	-2
Other cancers	410 902	448 886	0.92	-37 984	-11	-449 517	-5
Influenza and pneumonia	63 728	106 898	0.60	-43 170	-12	-324 863	-4
All other causes	191 851	168 165	1.14	23 686	7	1 262 110	15

Abbreviations: YLL = Years of life lost (summed across excess US deaths)
Notes: The counterfactual refers to the expected number of deaths the US would have experienced if it had death rates equal to the average of 17 other high-income countries, which are described in eTable 1.

© 2026 Bor J et al. *JAMA Network Open*.

eTable 6. Excess deaths and excess YLL in the US compared with other high-income countries by cause of death in 2009

				Excess US deaths		Excess US YLL	
Causes of death	Observed US deaths	Counterfactual deaths	Ratio of observed to counterfactual	No.	% of total	No.	% of total
All-cause	2 437 685	2 041 754	1.19	395 931	100	10 607 014	100
Circulatory	784 381	658 754	1.19	125 627	32	2 756 622	26
Mental and nervous system disorders	234 295	119 384	1.96	114 911	29	1 044 048	10
Respiratory	184 972	115 864	1.60	69 108	17	1 140 640	11
Diabetes, renal, and metabolic	136 349	83 111	1.64	53 238	13	1 093 974	10
Lung cancers	158 160	119 321	1.33	38 839	10	608 464	6
Drug poisoning	37 002	8 516	4.35	28 486	7	1 116 408	11
Infectious & parasitic	60 161	34 644	1.74	25 517	6	528 167	5
Transport accidents	39 184	18 195	2.15	20 989	5	841 446	8
Homicide	16 828	2 118	7.95	14 710	4	700 856	7
HIV/AIDS	9 408	2 057	4.57	7 351	2	243 754	2
Alcohol-related	24 524	23 845	1.03	679	0	63 118	1
Suicide	31 811	35 377	0.90	-3 566	-1	-44 428	0
Symptoms, signs, and ill-defined conditions	39 733	68 929	0.58	-29 196	-7	-139 921	-1
Influenza and pneumonia	53 707	86 172	0.62	-32 465	-8	-110 330	-1
Other cancers	424 149	477 921	0.89	-53 772	-14	-566 457	-5
All other causes	203 021	187 547	1.08	15 474	4	1 330 652	13

Abbreviations: YLL = Years of life lost (summed across excess US deaths)
Notes: The counterfactual refers to the expected number of deaths the US would have experienced if it had death rates equal to the average of 17 other high-income countries, which are described in eTable 1.

© 2026 Bor J et al. *JAMA Network Open*.

eTable 7. Excess deaths and excess YLL in the US compared with other high-income countries by cause of death in 2019

				Excess US deaths		Excess US YLL	
Causes of death	Observed US deaths	Counterfactual deaths	Ratio of observed to counterfactual	No.	% of total	No.	% of total
All-cause	2 854 691	2 218 385	1.29	636 306	100	16 088 339	100
Circulatory	858 666	592 955	1.45	265 711	42	4 800 773	30
Mental and nervous system disorders	356 210	203 042	1.75	153 168	24	1 780 260	11
Diabetes, renal, and metabolic	175 627	87 764	2.00	87 863	14	1 791 106	11
Respiratory	221 236	137 679	1.61	83 557	13	1 456 953	9
Drug poisoning	70 622	11 746	6.01	58 876	9	2 330 913	14
Transport accidents	41 947	12 621	3.32	29 326	5	1 130 684	7
Infectious & parasitic	59 661	36 197	1.65	23 464	4	552 144	3
Homicide	18 998	1 781	10.67	17 217	3	819 952	5
Alcohol-related	39 034	24 418	1.60	14 616	2	487 160	3
Suicide	42 684	31 849	1.34	10 835	2	469 746	3
Lung cancers	139 680	131 352	1.06	8 328	1	129 638	1
HIV/AIDS	5 042	907	5.56	4 135	1	125 184	1
Influenza and pneumonia	49 780	78 918	0.63	-29 138	-5	-163 254	-1
Other cancers	475 545	527 188	0.90	-51 643	-8	-433 504	-3
Symptoms, signs, and ill-defined conditions	47 671	125 703	0.38	-78 032	-12	-882 936	-5
All other causes	252 288	214 266	1.18	38 022	6	1 693 518	11

Abbreviations: YLL = Years of life lost (summed across excess US deaths)
Notes: Notes: The counterfactual refers to the expected number of deaths the US would have experienced if it had death rates equal to the average of 17 other high-income countries, which are described in eTable 1.

© 2026 Bor J et al. *JAMA Network Open*.

eTable 8. Excess deaths and excess YLL in the US compared with other high-income countries by cause of death in 2020

				Excess US deaths		Excess US YLL	
Causes of death	Observed US deaths	Counterfactual deaths	Ratio of observed to counterfactual	No.	% of total	No.	% of total
All-cause	3 383 613	2 375 470	1.42	1 008 143	100	22 760 636	100
Circulatory	911 999	595 468	1.53	316 531	31	5 521 057	24
Mental and nervous system disorders	395 329	203 493	1.94	191 836	19	2 138 488	9
COVID-19	350 827	162 633	2.16	188 194	19	3 161 630	14
Diabetes, renal, and metabolic	197 276	92 992	2.12	104 284	10	2 078 195	9
Respiratory	216 957	130 516	1.66	86 441	9	1 483 711	7
Drug poisoning	91 783	12 921	7.10	78 862	8	3 125 902	14
Transport accidents	45 159	10 893	4.15	34 266	3	1 343 167	6
Infectious & parasitic	63 199	33 714	1.87	29 485	3	642 379	3
Homicide	24 406	1 765	13.83	22 641	2	1 072 892	5
Alcohol-related	49 057	26 667	1.84	22 390	2	716 162	3
Suicide	41 604	32 267	1.29	9 337	1	424 551	2
Lung cancers	136 166	130 645	1.04	5 521	1	85 995	0
HIV/AIDS	5 115	891	5.74	4 224	0	124 768	1
Influenza and pneumonia	53 542	63 637	0.84	-10 095	-1	97 181	0
Other cancers	482 454	527 925	0.91	-45 471	-5	-316 661	-1
Symptoms, signs, and ill-defined conditions	50 098	131 610	0.38	-81 512	-8	-858 558	-4
All other causes	268 642	217 431	1.24	51 211	5	1 919 776	8

Abbreviations: YLL = Years of life lost (summed across excess US deaths)
Notes: Notes: The counterfactual refers to the expected number of deaths the US would have experienced if it had death rates equal to the average of 17 other high-income countries, which are described in eTable 1.

© 2026 Bor J et al. *JAMA Network Open*.

eTable 9. Excess deaths and excess YLL in the US compared with other high-income countries by cause of death in 2021

				Excess US deaths		Excess US YLL	
Causes of death	Observed US deaths	Counterfactual deaths	Ratio of observed to counterfactual	No.	% of total	No.	% of total
All-cause	3 464 138	2 289 852	1.51	1 174 286	100	27 437 210	100
Circulatory	930 845	547 311	1.70	383 534	33	6 511 369	24
COVID-19	416 890	145 616	2.86	271 274	23	5 562 600	20
Mental and nervous system disorders	370 791	192 152	1.93	178 639	15	2 001 678	7
Diabetes, renal, and metabolic	203 959	86 663	2.35	117 296	10	2 314 849	8
Drug poisoning	106 689	15 243	7.00	91 446	8	3 597 890	13
Respiratory	208 916	128 401	1.63	80 515	7	1 455 789	5
Transport accidents	49 841	11 429	4.36	38 412	3	1 503 683	5
Alcohol-related	54 255	23 839	2.28	30 416	3	941 784	3
Infectious & parasitic	63 584	33 556	1.89	30 028	3	679 187	2
Homicide	25 816	1 774	14.55	24 042	2	1 142 991	4
Suicide	43 745	33 258	1.32	10 487	1	453 536	2
Lung cancers	134 590	129 468	1.04	5 122	0	118 908	0
HIV/AIDS	4 977	825	6.03	4 152	0	124 053	0
Influenza and pneumonia	41 916	55 406	0.76	-13 490	-1	34 628	0
Other cancers	486 916	521 466	0.93	-34 550	-3	-130 399	0
Symptoms, signs, and ill-defined conditions	34 487	144 518	0.24	-110 031	-9	-1 146 269	-4
All other causes	285 921	218 928	1.31	66 993	6	2 270 932	8

Abbreviations: YLL = Years of life lost (summed across excess US deaths)
Notes: The counterfactual refers to the expected number of deaths the US would have experienced if it had death rates equal to the average of 17 other high-income countries, which are described in eTable 1.

© 2026 Bor J et al. *JAMA Network Open*.

eTable 10. Changes in excess US deaths and excess US YLL by cause of death from 1999 to 2019, 2019 to 2022, and 1999 to 2022

	Change in excess US deaths						Change in excess US YLL					
	1999-2019	% of total	2019-2022	% of total	1999-2022	% of total	1999-2019	% of total	2019-2022	% of total	1999-2022	% of total
All-cause	290 139	100	268 853	100	558 992	100	7 747 192	100	5 795 703	100	13 542 895	100
Circulatory	97 722	34	92 862	35	190 584	34	2 059 980	27	1 119 442	19	3 179 423	23
Mental and nervous system disorders	100 055	34	25 948	10	126 002	23	1 289 558	17	182 869	3	1 472 427	11
Diabetes, renal, and metabolic	40 410	14	26 675	10	67 085	12	937 668	12	430 286	7	1 367 954	10
Drug poisoning	47 789	16	34 627	13	82 416	15	1 913 883	25	1 284 110	22	3 197 993	24
COVID-19	0	0	93 291	35	93 291	17	0	0	1 734 445	30	1 734 445	13
Respiratory	26 621	9	-13 475	-5	13 146	2	605 782	8	-155 497	-3	450 284	3
All other causes	14 336	5	14 300	5	28 636	5	431 408	6	347 082	6	778 490	6
Transport accidents	11 420	4	7 841	3	19 261	3	379 191	5	299 720	5	678 911	5
Alcohol-related	16 533	6	12 378	5	28 910	5	522 707	7	350 350	6	873 057	6
Infectious & parasitic	7 345	3	2 995	1	10 340	2	259 653	3	60 764	1	320 417	2
Homicide	2 960	1	5 694	2	8 654	2	152 541	2	259 682	4	412 223	3
Suicide	25 769	9	-183	0	25 586	5	843 028	11	-29 506	-1	813 522	6
HIV/AIDS	-8 049	-3	4	0	-8 045	-1	-311 434	-4	-3 476	0	-314 910	-2
Lung cancers	-46 760	-16	-7 809	-3	-54 570	-10	-768 786	-10	-73 009	-1	-841 794	-6
Influenza and pneumonia	14 032	5	-395	0	13 637	2	161 609	2	38 778	1	200 387	1
Other cancers	-13 658	-5	12 591	5	-1 067	0	16 013	0	277 898	5	293 911	2
Symptoms, signs, and ill-defined conditions	-46 383	-16	-38 491	-14	-84 874	-15	-745 610	-10	-328 237	-6	-1 073 846	-8

Abbreviations: YLL = Years of life lost (summed across excess US deaths)
Notes: Excess US deaths refer to the difference between observed US deaths and deaths expected if US mortality rates were equal to the average of the 17 other high-income countries.

© 2026 Bor J et al. *JAMA Network Open*.

eTable 11. Age distribution of excess US deaths and excess US YLL by cause of death in 2022

	Excess US Deaths								Excess US YLL							
	0-24	25-34	35-44	45-54	55-64	65-74	75-84	85+	0-24	25-34	35-44	45-54	55-64	65-74	75-84	85+
All-cause	35 285	51 012	69 346	92 804	185 522	204 433	181 584	85 173	2 464 078	2 648 226	2 946 294	3 077 945	4 599 694	3 526 684	1 963 178	657 943
Homicide	6 867	6 333	4 469	2 476	1 623	743	308	91	432 210	325 164	186 776	80 185	39 047	12 352	3 214	688
Transport accidents	6 406	7 074	6 129	5 178	5 604	3 820	2 124	831	408 948	367 127	258 432	169 481	135 463	63 023	21 864	6 067
Drug poisoning	5 982	20 402	24 065	19 350	17 877	5 450	457	-79	374 749	1 058 451	1 017 938	635 600	434 346	89 865	4 667	-591
Suicide	2 334	3 029	1 881	995	1 041	428	760	185	141 876	154 123	77 351	30 691	23 164	5 128	6 850	1 057
Circulatory	837	2 964	9 834	23 279	60 882	84 363	85 590	90 824	58 355	154 948	419 621	771 029	1 497 255	1 431 647	906 689	680 672
Respiratory	609	578	1 091	3 009	14 613	24 594	23 628	1 960	44 739	30 429	47 267	102 966	369 231	427 615	258 573	20 636
COVID-19	560	1 323	3 198	7 776	17 822	25 794	23 778	13 039	39 152	69 305	137 203	259 158	442 896	439 200	250 641	96 890
Infectious & parasitic	552	508	1 209	2 947	6 974	9 445	5 878	-1 055	43 407	26 805	52 012	98 868	174 388	162 694	62 620	-7 886
Mental and nervous system disorders	471	1 142	1 650	2 098	5 415	16 388	49 533	102 419	29 255	59 875	70 966	70 671	135 627	284 204	532 553	779 977
Diabetes, renal, and metabolic	437	1 886	4 570	10 620	23 610	34 286	28 909	10 219	28 125	98 860	195 260	352 793	583 086	582 958	304 870	75 440
Influenza and pneumonia	226	298	619	904	2 089	-21	-8 551	-25 098	17 622	15 708	26 603	30 455	53 108	3 219	-86 687	-184 504
Other cancers	209	520	1 456	2 761	5 847	-9 234	-21 652	-18 958	12 314	27 096	63 264	91 334	149 246	-142 084	-218 913	-137 865
Alcohol-related	155	2 393	5 139	6 199	8 815	3 546	581	167	9 550	124 542	217 920	204 266	215 207	58 760	6 030	1 234
HIV/AIDS	37	433	658	814	1 134	784	234	45	2 278	22 307	27 691	26 579	27 305	12 875	2 357	315
Lung cancers	-9	9	-185	-136	2 806	-2 090	540	-418	-587	489	-7 822	-3 209	76 852	-22 925	14 398	-567
Symptoms, signs, and ill-defined conditions	587	-1 115	-1 674	-2 761	-5 308	-9 351	-17 242	-79 658	72 187	-57 557	-69 682	-89 145	-126 081	-153 671	-181 616	-605 609
All other causes	9 025	3 235	5 238	7 293	14 677	15 490	6 708	-9 342	749 897	170 555	225 492	246 223	369 553	271 824	75 069	-68 012

Abbreviations: YLL = Years of life lost (summed across excess US deaths)
Notes: Excess US deaths refer to the difference between observed US deaths and deaths expected if US mortality rates were equal to the average of the 17 other high-income countries.

© 2026 Bor J et al. *JAMA Network Open*.

eTable 12. Excess US deaths and excess US YLL by sex and cause of death from 1999 to 2022

				Excess US deaths		Excess US YLL	
	Observed deaths	Counterfactual deaths	Ratio of observed to counterfactual	No.	% of total	No.	% of total
Male							
All-cause	32 041 864	26 556 109	1.21	5 485 755	100	157 238 344	100
Circulatory	10 153 705	7 668 538	1.32	2 485 167	45	46 097 242	29
Mental and nervous system disorders	2 326 935	1 381 740	1.68	945 195	17	9 618 692	6
Diabetes, renal, and metabolic	1 850 040	965 595	1.92	884 445	16	16 580 951	11
Drug poisoning	748 311	138 039	5.42	610 272	11	23 488 682	15
Respiratory	2 260 598	1 690 025	1.34	570 573	10	9 944 643	6
Transport accidents	748 155	333 645	2.24	414 510	8	15 923 996	10
Homicide	351 984	31 384	11.22	320 600	6	15 036 690	10
COVID-19	531 775	229 380	2.32	302 395	6	5 661 006	4
Infectious & parasitic	675 286	409 397	1.65	265 889	5	5 652 408	4
HIV/AIDS	162 676	35 031	4.64	127 645	2	4 040 198	3
Alcohol-related	520 797	434 549	1.20	86 248	2	3 095 285	2
Suicide	686 302	626 195	1.10	60 107	1	3 580 193	2
Lung cancers	2 024 595	1 967 859	1.03	56 736	1	812 539	1
Influenza and pneumonia	629 131	1 055 944	0.60	-426 813	-8	-2 636 380	-2
Symptoms, signs, and ill-defined conditions	398 001	927 385	0.43	-529 384	-10	-5 962 269	-4
Other cancers	5 406 137	6 339 271	0.85	-933 134	-17	-10 622 698	-7
All other causes	2 567 436	2 322 133	1.11	245 303	4	16 927 166	11
Female							
All-cause	31 505 454	24 315 563	1.30	7 189 891	100	157 068 807	100
Circulatory	10 443 060	8 152 464	1.28	2 290 596	32	41 615 332	26
Mental and nervous system disorders	3 886 262	1 948 060	1.99	1 938 202	27	19 237 066	12
Respiratory	2 365 481	1 225 643	1.93	1 139 838	16	18 250 491	12
Diabetes, renal, and metabolic	1 783 733	1 020 183	1.75	763 550	11	15 737 272	10
Lung cancers	1 624 505	913 056	1.78	711 449	10	11 352 375	7
Drug poisoning	398 504	86 350	4.61	312 154	4	12 956 668	8
Infectious & parasitic	711 769	410 734	1.73	301 035	4	6 286 125	4
COVID-19	422 493	172 129	2.45	250 364	3	4 797 669	3
Transport accidents	307 804	111 389	2.76	196 415	3	8 453 757	5
Homicide	93 264	17 106	5.45	76 158	1	3 732 586	2
HIV/AIDS	57 984	9 730	5.96	48 254	1	1 843 596	1
Alcohol-related	190 470	142 443	1.34	48 027	1	1 896 672	1
Suicide	137 957	202 542	0.68	-64 585	-1	-1 463 154	-1
Other cancers	5 159 798	5 409 171	0.95	-249 373	-3	-1 112 987	-1
Influenza and pneumonia	716 908	970 544	0.74	-253 636	-4	-578 161	0
Symptoms, signs, and ill-defined conditions	461 989	1 176 410	0.39	-714 421	-10	-5 048 991	-3
All other causes	2 743 473	2 347 609	1.17	395 864	6	19 112 491	12

Abbreviations: YLL = Years of life lost (summed across excess US deaths)
Notes: Excess US deaths refer to the difference between observed US deaths and deaths expected if US mortality rates were equal to the average of the 17 other high-income countries.

eFigure 1. Annual observed deaths in the US, counterfactual deaths in other high-income countries, and excess deaths in the US compared with other high-income countries for each cause of death from 1999 to 2022

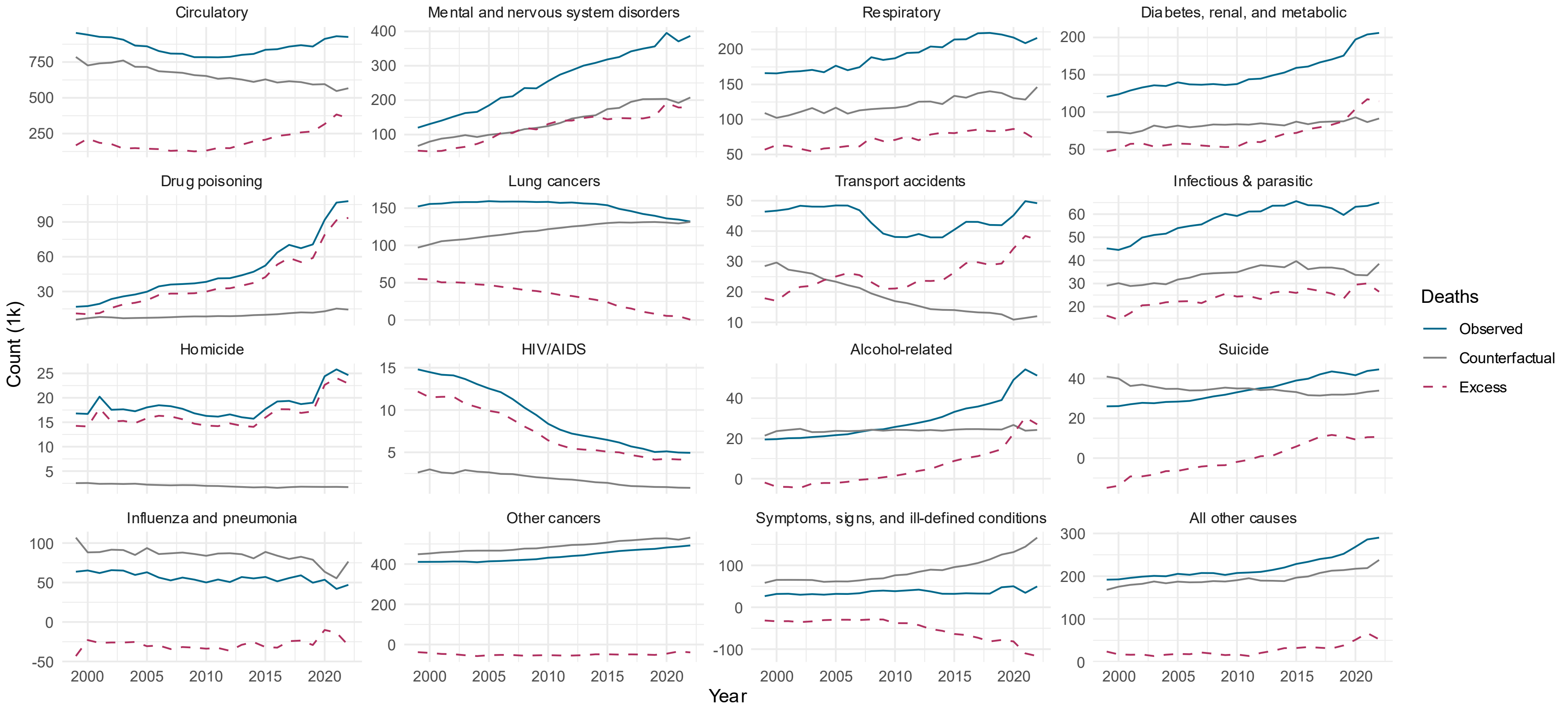

Notes: The counterfactual refers to the expected number of deaths the US would have experienced if it had death rates equal to the average of 17 other high-income countries, which are described in eTable 1.

© 2026 Bor J et al. *JAMA Network Open*.

eFigure 2. Annual observed YLL in the US, counterfactual YLL in other high-income countries, and excess YLL in the US compared with other high-income countries for each cause of death from 1999 to 2022

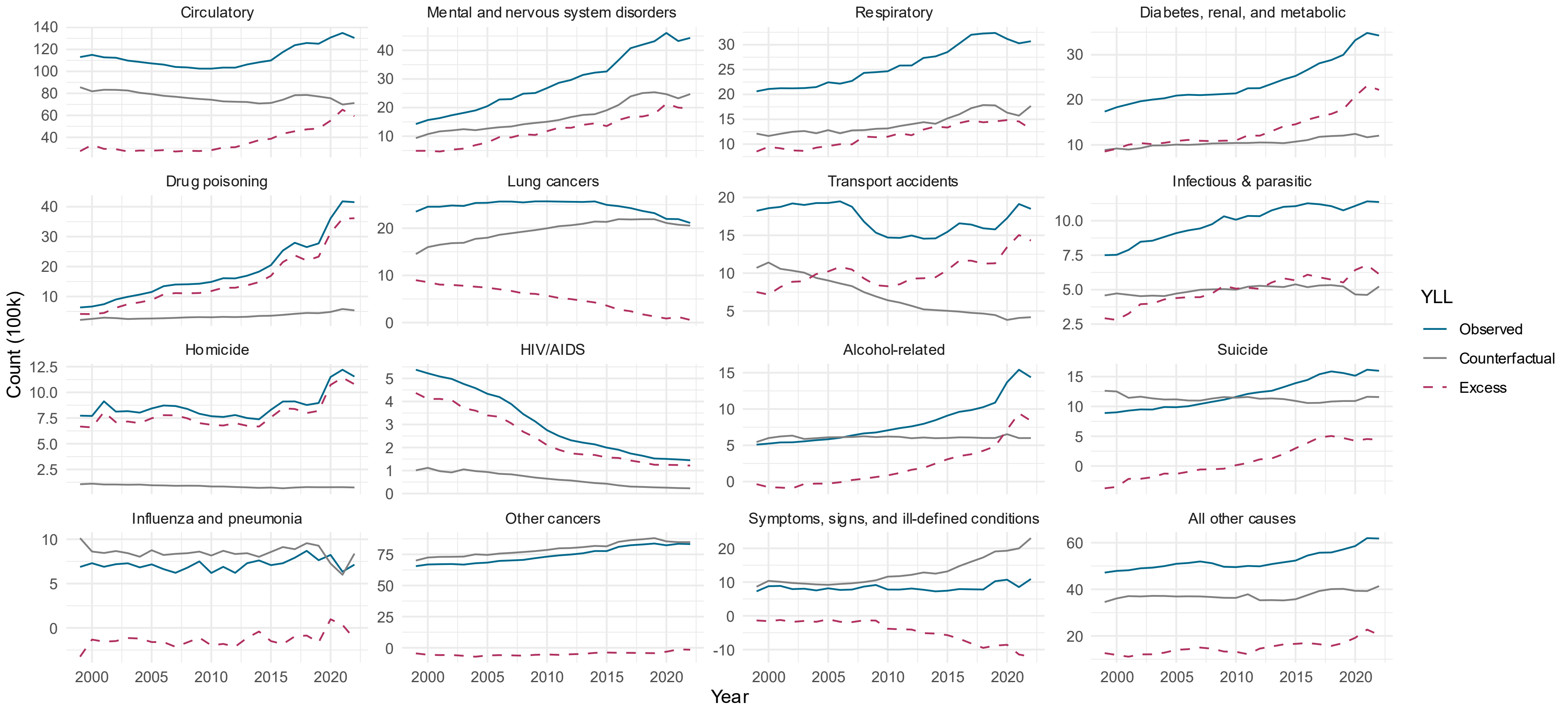

Abbreviations: YLL = Years of life lost (summed across excess US deaths)
Notes: The counterfactual refers to the expected number of YLL the US would have experienced if it had death rates equal to the average of 17 other high-income countries, which are described in eTable 1.

© 2026 Bor J et al. *JAMA Network Open*.

eFigure 3. Rank order of excess US deaths by cause from 1999 to 2022

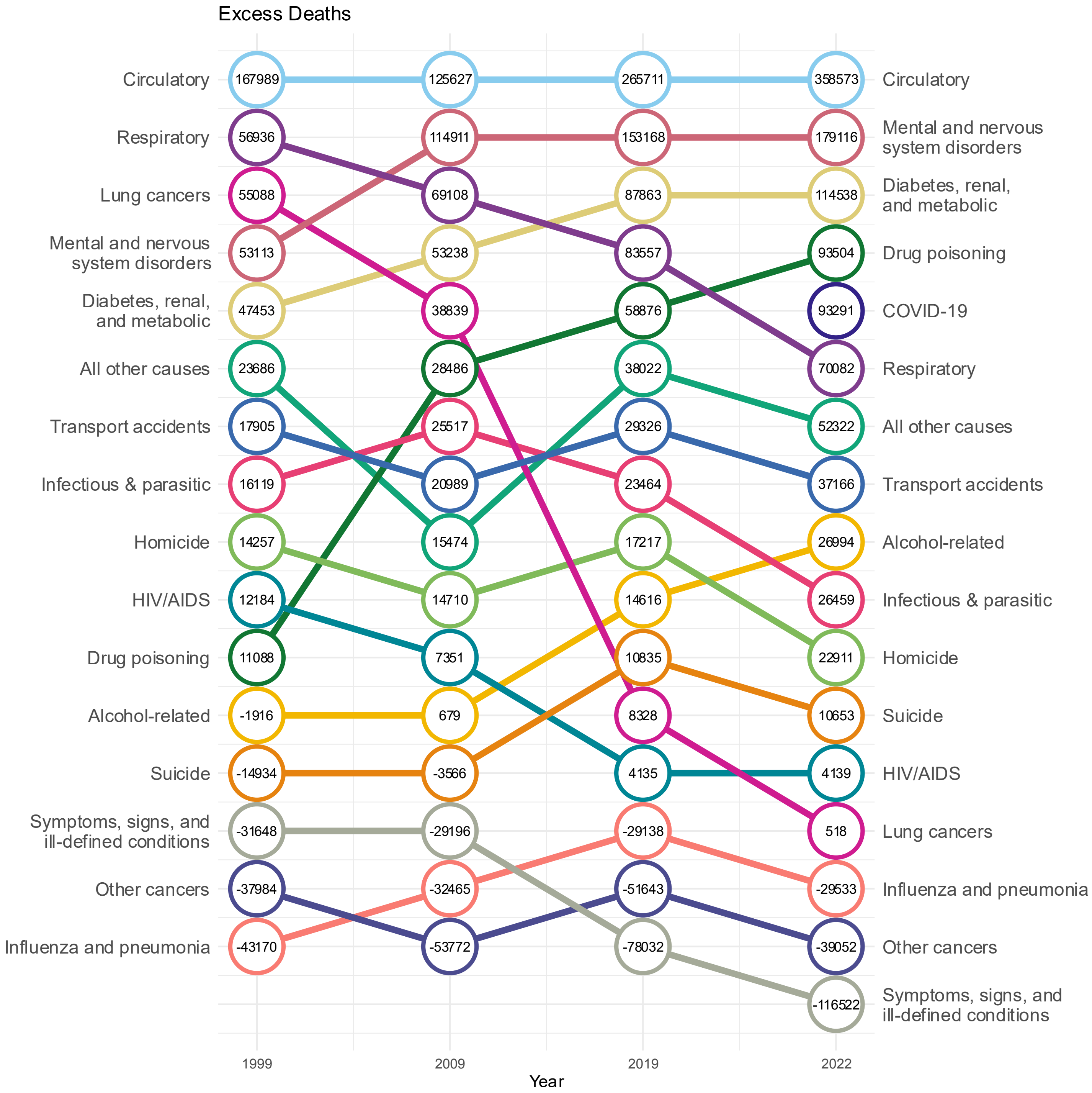

Notes: Excess US deaths refer to the difference between observed US deaths and deaths expected if US mortality rates were equal to the average of the 17 other high-income countries.

© 2026 Bor J et al. *JAMA Network Open*.

eFigure 4. Change in slope across time periods for top 4 causes of excess US deaths

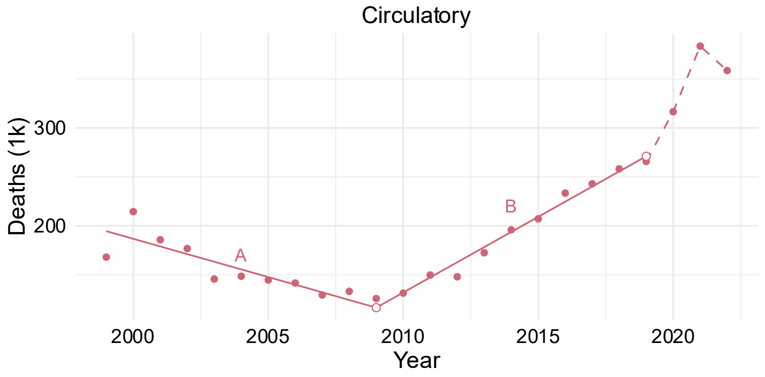

Segment	Years	Annualized rate of change (95% CI) (1k excess deaths)	P value
A	1999-2009	−7.8 (−10.1, −5.5)	
B	2009-2019	15.5 (13.5, 17.4)	<0.001

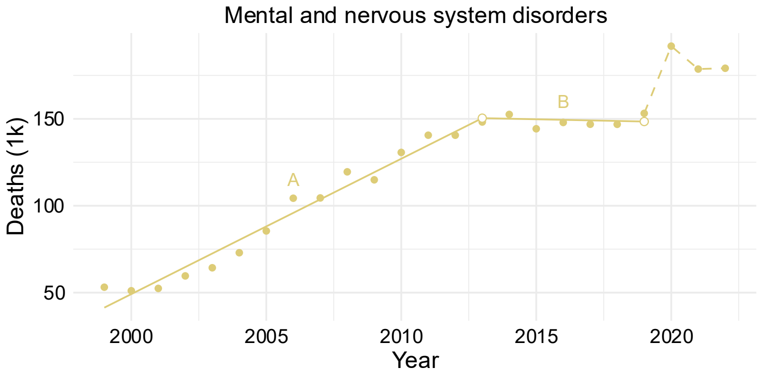

Segment	Years	Annualized rate of change (95% CI) (1k excess deaths)	P value
A	1999-2013	7.8 (6.9, 8.6)	
B	2013-2019	−0.3 (−3.6, 3.0)	<0.001

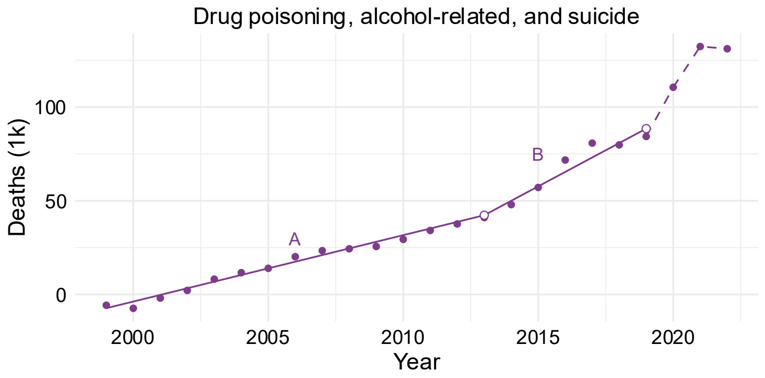

Segment	Years	Annualized rate of change (95% CI) (1k excess deaths)	P value
A	1999-2013	3.5 (3.0, 4.1)	
B	2013-2019	7.7 (6.0, 9.4)	<0.001

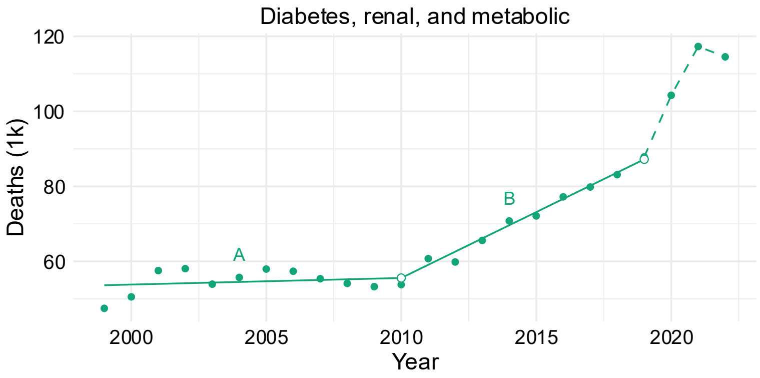

Segment	Years	Annualized rate of change (95% CI) per 1k	P value
A	1999-2010	0.2 (−0.4, 0.8)	
B	2010-2019	3.5 (2.8, 4.2)	<0.001

Notes: Excess US deaths refer to the difference between observed US deaths and deaths expected if US mortality rates were equal to the average of the 17 other high-income countries. The P values indicate whether the change in slope between segments A and B was significant.

© 2026 Bor J et al. *JAMA Network Open*.

eFigure 5. Regression analysis of prepandemic trends of top 4 causes of excess US deaths

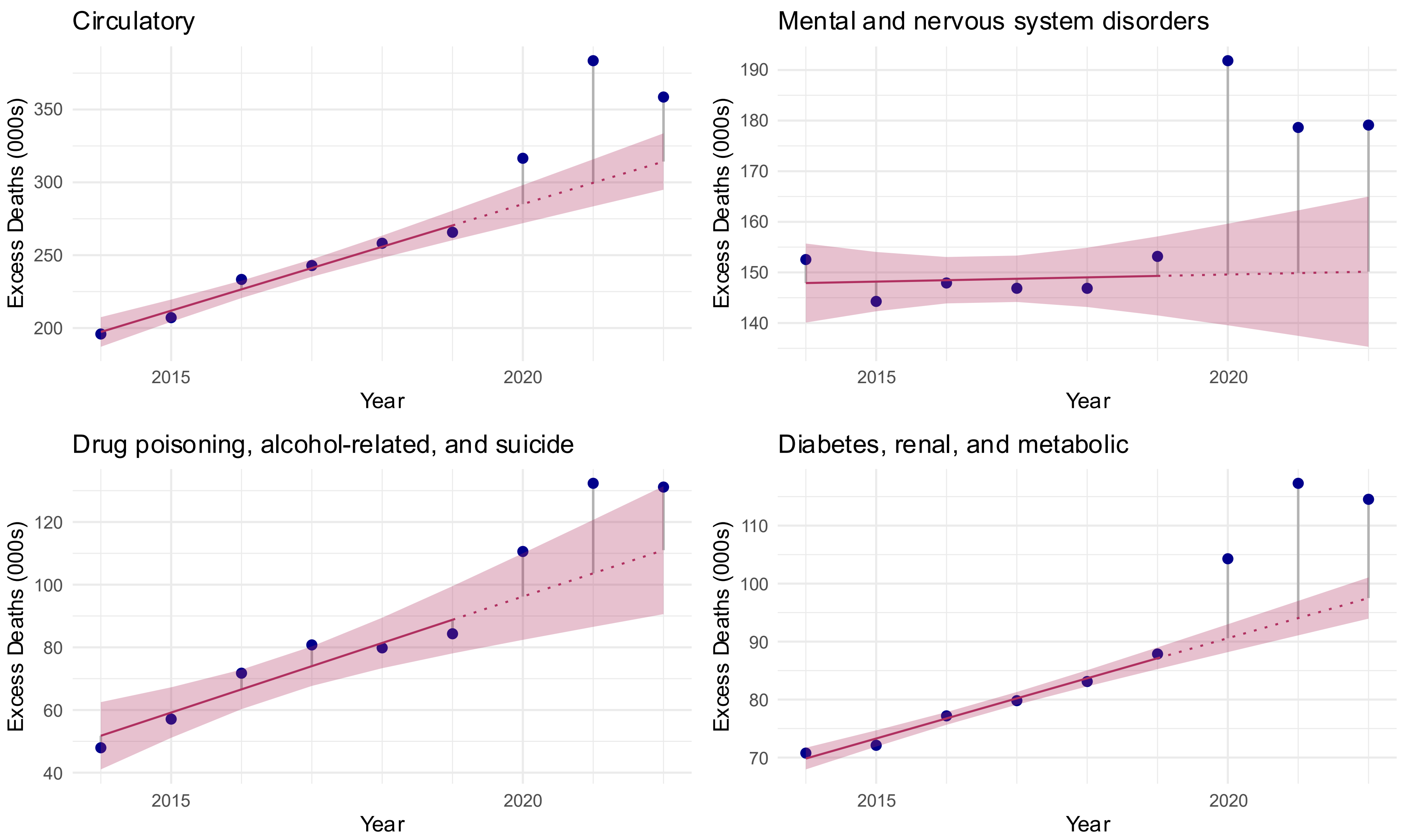

Notes: Excess US deaths refer to the difference between observed US deaths and deaths expected if US mortality rates were equal to the average of the 17 other high-income countries. The dotted red line represents the anticipated number of excess US deaths in 2020, 2021, and 2022 based on pre-pandemic trends in excess deaths from 2014 to 2019. The shaded red region represents the 95% confidence interval for these estimates, which were generated using linear regression. The solid black dots reflect the actual number of excess deaths. When the solid black dots in 2020, 2021, and/or 2022 are above the red region, this indicates that excess US deaths in these years exceeded the number of excess US deaths anticipated based on pre-pandemic trends.

© 2026 Bor J et al. *JAMA Network Open*.

eFigure 6. Annual excess US deaths by age group and cause of death from 1999 to 2022

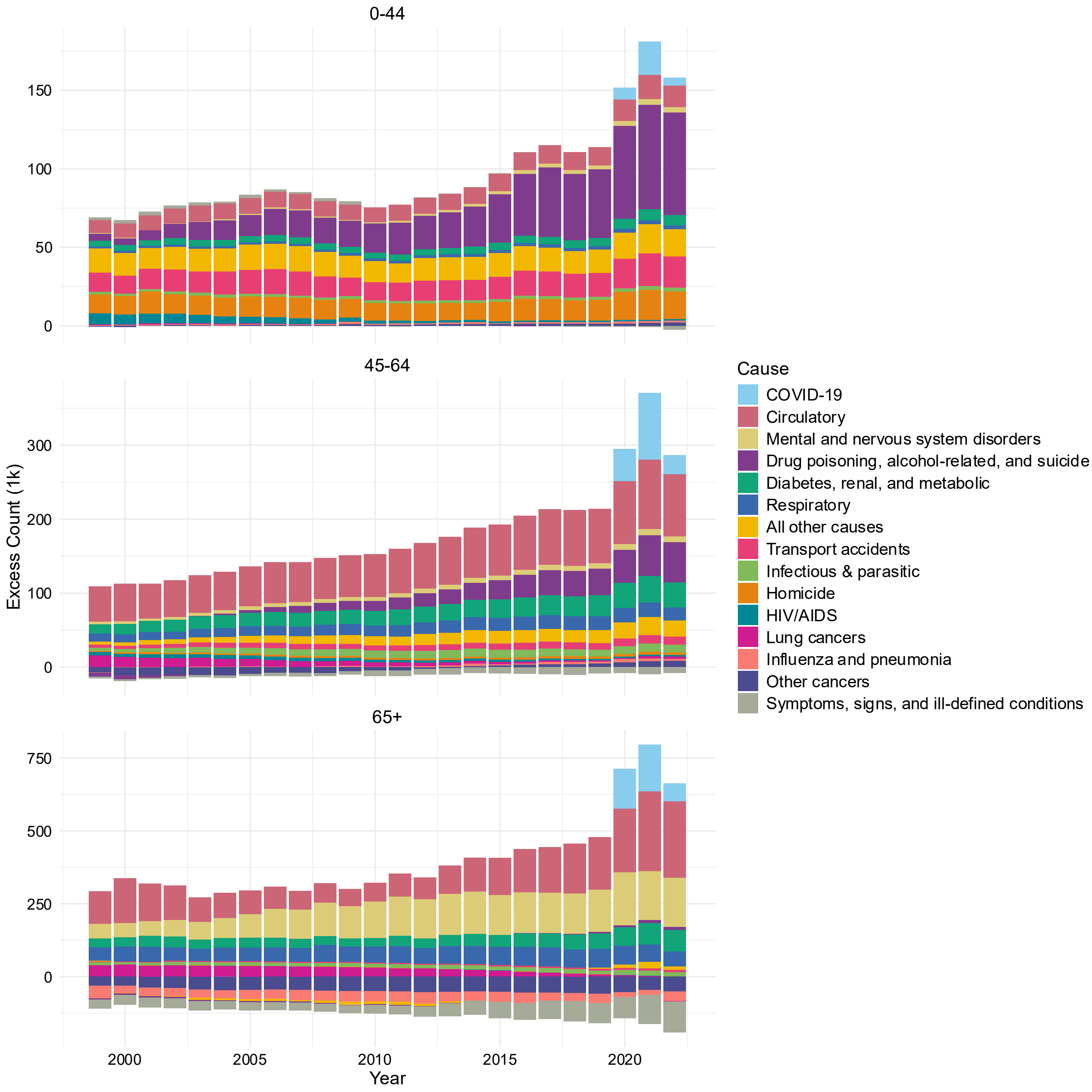

Notes: Excess US deaths refer to the difference between observed US deaths and deaths expected if US mortality rates were equal to the average of the 17 other high-income countries.

© 2026 Bor J et al. *JAMA Network Open*.

eFigure 7. Annual excess US deaths and excess US YLL by age group and cause of death from 1999 to 2022

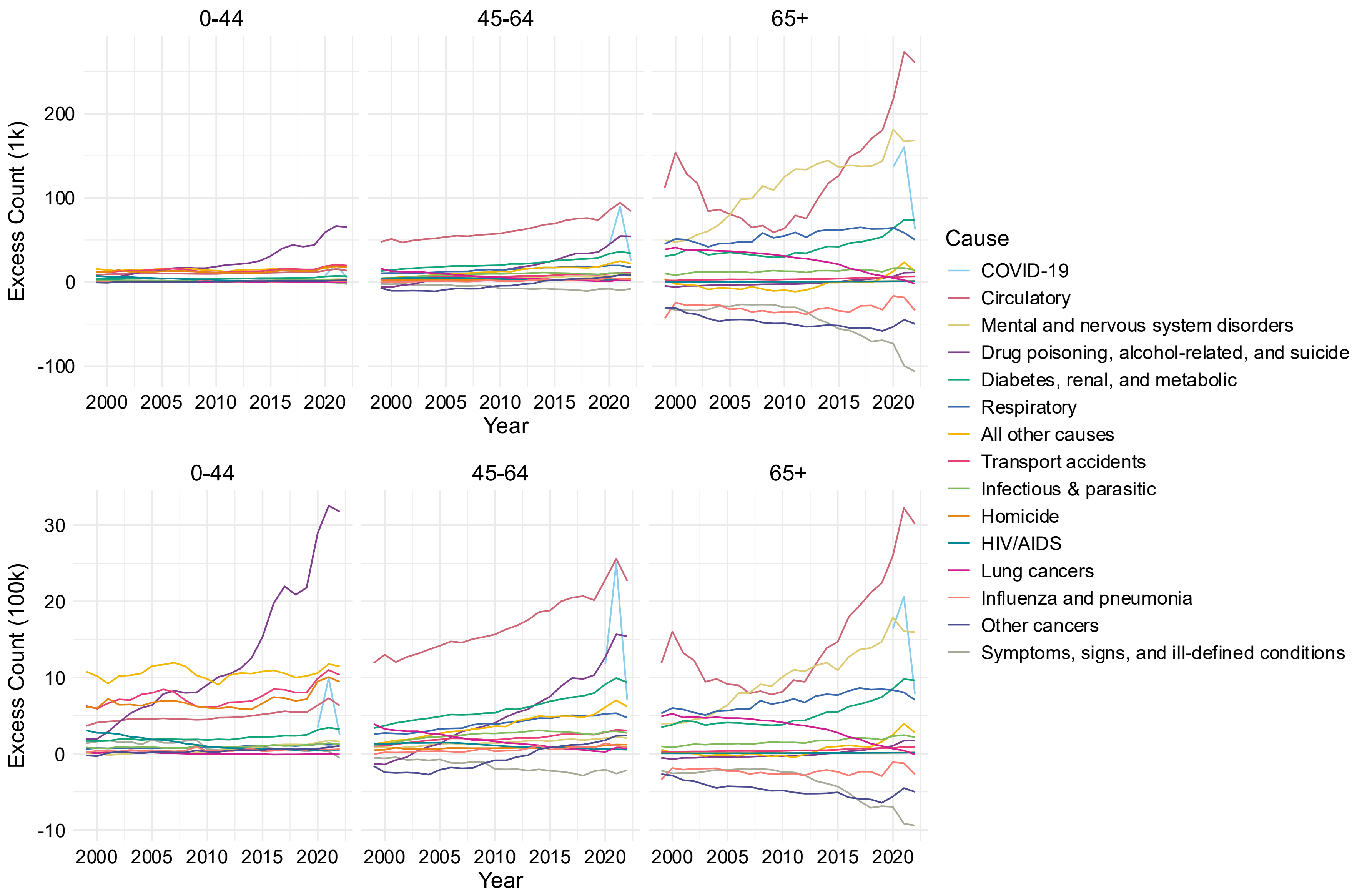

Abbreviations: YLL = Years of life lost (summed across excess US deaths)
Notes: Excess US deaths are reported in the top row, and excess US YLL in the bottom. Excess US deaths refer to the difference between observed US deaths and deaths expected if US mortality rates were equal to the average of the 17 other high-income countries.

© 2026 Bor J et al. *JAMA Network Open*.

eFigure 8. Annual excess US deaths and excess US YLL by sex and cause of death from 1999 to 2022

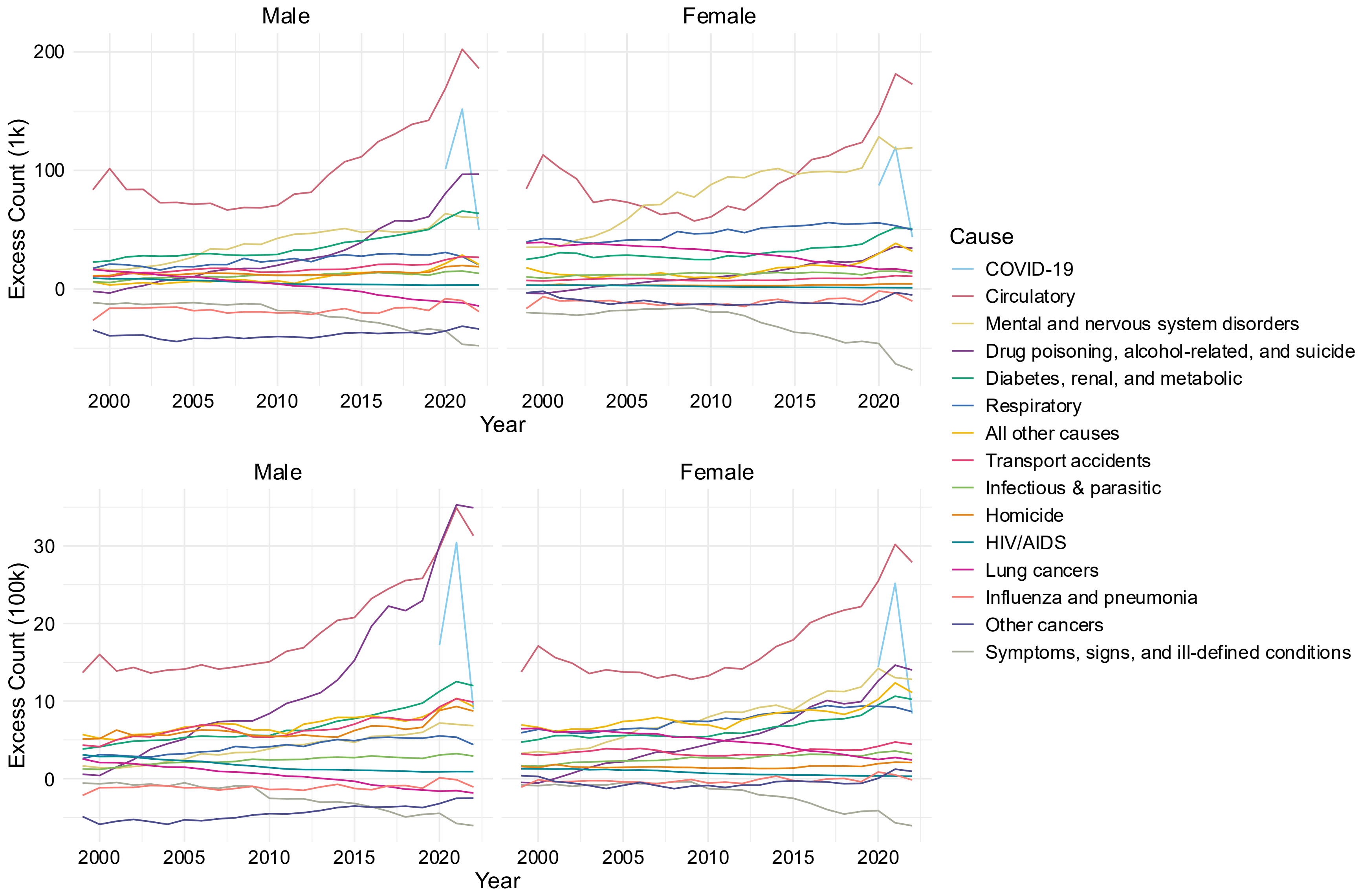

Abbreviations: YLL = Years of life lost (summed across excess US deaths)
Notes: Excess US deaths are reported in the top row, and excess US YLL in the bottom. Excess US deaths refer to the difference between observed US deaths and deaths expected if US mortality rates were equal to the average of the 17 other high-income countries.

© 2026 Bor J et al. *JAMA Network Open*.

eAppendix. Supplementary Data & Replication Code

The underlying data used in the study are publicly available from the World Health Organization and the Human Mortality Database. The estimates generated in this study and programming code for replicating the analyses can be downloaded from the following permanent repository: https://osf.io/85sk2/

© 2026 Bor J et al. *JAMA Network Open*.
