# Supplementary material for: Causes of Excess Deaths in the US Compared With Other High-Income Countries
Source: JAMA Netw Open. 2026 May 8;9(5):e266147. doi: 10.1001/jamanetworkopen.2026.6147 (PMC13156795; doi:10.1001/jamanetworkopen.2026.6147)
Supplement: Supplement 2. — Data Sharing Statement [file jamanetwopen-e266147-s002.pdf]

Data Sharing Statement

Bor. Causes of Excess Deaths in the US Compared With Other High-Income Countries. *JAMA Netw Open*. Published May 08, 2026. doi:10.1001/jamanetworkopen.2026.6147

Data
Data available: Yes

Additional Information
Data types: Data (not involving human participants) and analytic code
How to access data: The underlying data used in the study are publicly available from the World Health Organization and the Human Mortality Database. The estimates generated in this study and programming code for replicating the analyses can be downloaded from the following permanent repository: https://osf.io/85sk2/
When available: With publication
